# Supplementary material for: Quantifying reference alignment bias in functional genomics analyses
Source: Cell Rep Methods. 2026 May 14;6(7):101461. doi: 10.1016/j.crmeth.2026.101461 (PMC13390086; doi:10.1016/j.crmeth.2026.101461)
Supplement: Document S1. Figures S1–S7 [file mmc1.pdf]

Cell Reports Methods, Volume *6*

Supplemental information

Quantifying reference alignment bias

in functional genomics analyses

Nina Tekkey, John E. Garza, Wenjin Zhang, Derek Albracht, Chad Tomlinson, Edward A. Belter, Xiaoyun Xing, Juan F. Macias-Velasco, and Ting Wang

Supplement

Supplemental Table 1: Bin fate fractions as an average across all five individuals and replicates and fractions of the genome affected by RAB on a chromosome level, as an average across all samples, related to Figures 3 and 4. Left to right, columns are chromosome, parent, technology, reference, mean fraction of unequal bins, standard deviation of fraction of unequal bins, mean fraction of equal bins, standard deviation of fraction of equal bins, mean fraction of bins that did not liftover, standard deviation of fraction of bins that did not liftover, mean fraction of bins that did not have reads, standard deviation of fraction of bins that did not have reads, mean total number of bins, standard deviation of total number of bins, mean of total bins across parents, standard deviation of total bins, mean fraction of bins located on chromosome, mean fraction of equal bins, standard deviation of fraction of equal bins, mean fraction of unequal bins, standard deviation of fraction of unequal bins, mean fraction of equal bins with RAB, mean fraction of unequal bins with RAB, mean total fraction of genome affected by RAB per chromosome..

Supplemental Table 2: ANOVA results for all 3 technologies using the model: ks ~ individual+chromosome+individual:chromosome+replicate+Δbinlength, related to Figure 5. Columns from left to right are variables, degrees of freedom, sum squared, mean squared, F value, P value, P values adjusted by the Benjamini-Hochberg FDR method, technology, and reference.

Supplemental Table 3: Fractions of annotation space affected by RAB on a genome level, as an average across all samples, related to Figure 4. Left to right, columns are technology, annotation set, total number of annotations in set, fraction of annotations intersected with equal length bins, fraction of annotations intersected with unequal length bins, fraction of annotations intersected with equal length bins with RAB, fraction of annotations intersected with unequal length bins with RAB.

Supplemental Table 4: Genome Assembly Statistics, related to STAR Methods. Left to right, columns are assembly, number of contigs, N50, L50, number of single-copy genes, number of false duplications, fragment number, switch error rate, Hamming error rate, QV value, scaffold assembly gaps, and scaffold assembly gaps base pair size.

Supplemental Table 5: WebGestalt Over-representation analysis results, related to Figure 4. This table contains the raw results returned from WebGestalt after submitting RAB genes from each strata to each of several databases. Left to right, columns are stratum name, source database, an identified over-represented gene set, the description of the gene set, a link to the source of the gene set, the size of the set, the overlap between the submitted set and the identified set, the expected overlap, the enrichment ratio, the p-value, the false discovery rate (FDR), IDs of overlapping genes in the set, and the submitted gene IDs (ensembl gene IDs).

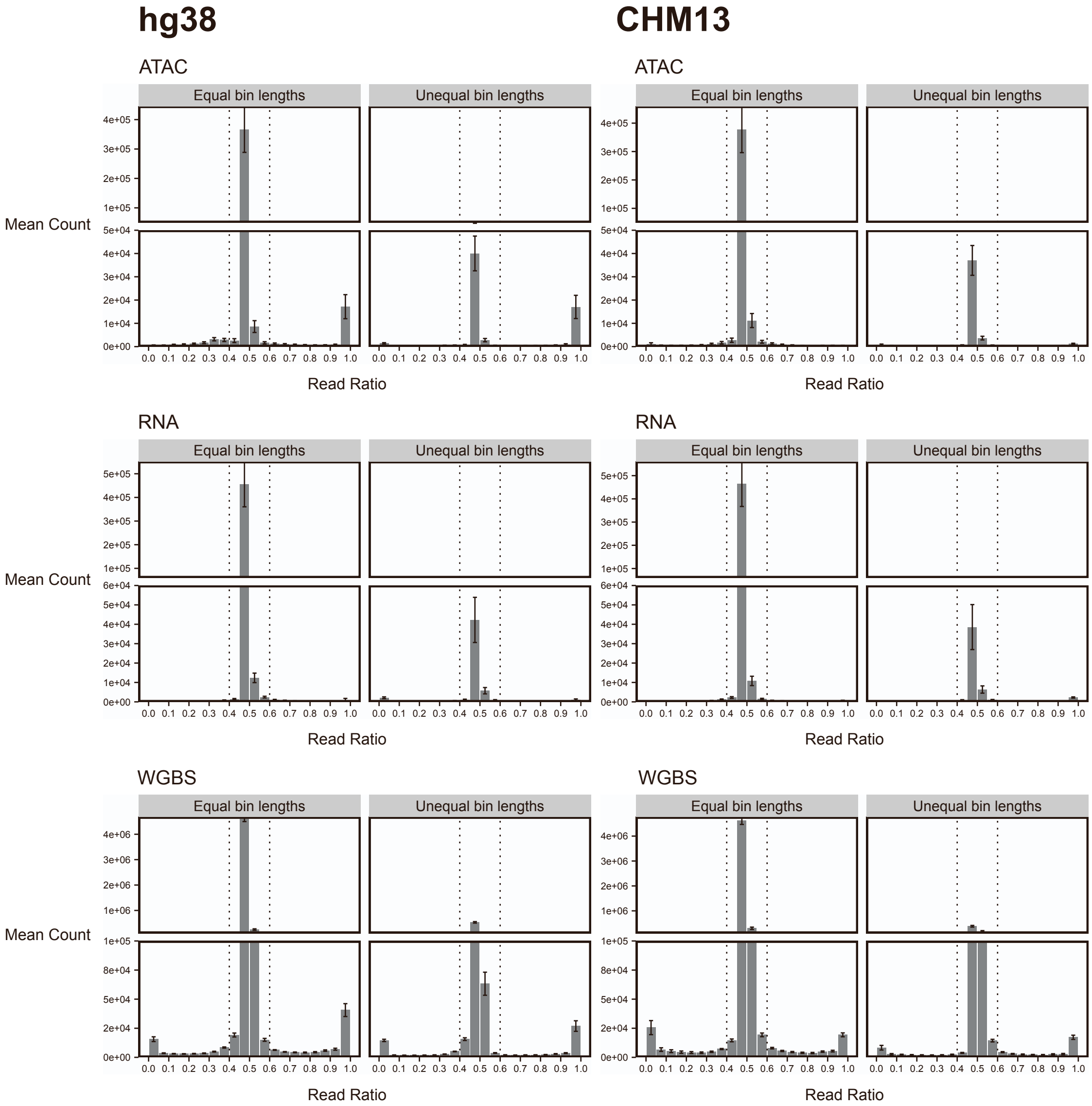

Supplemental Figure 1: <u>Distribution of reference fractions (Rf) across equal- and unequal-length bins for each sequencing technology and reference genome, related to Figure 4.</u> Each panel shows the count of bins (y-axis) as a function of reference fraction (Rf, x-axis), separated into equal- and unequal-length bins (left and right, respectively). Rows correspond to sequencing technologies (ATAC-seq, RNA-seq, and WGBS), and super-columns correspond to reference genomes (hg38 and CHM13). Dashed vertical lines indicate the Rf = 0.4 and 0.6 thresholds.

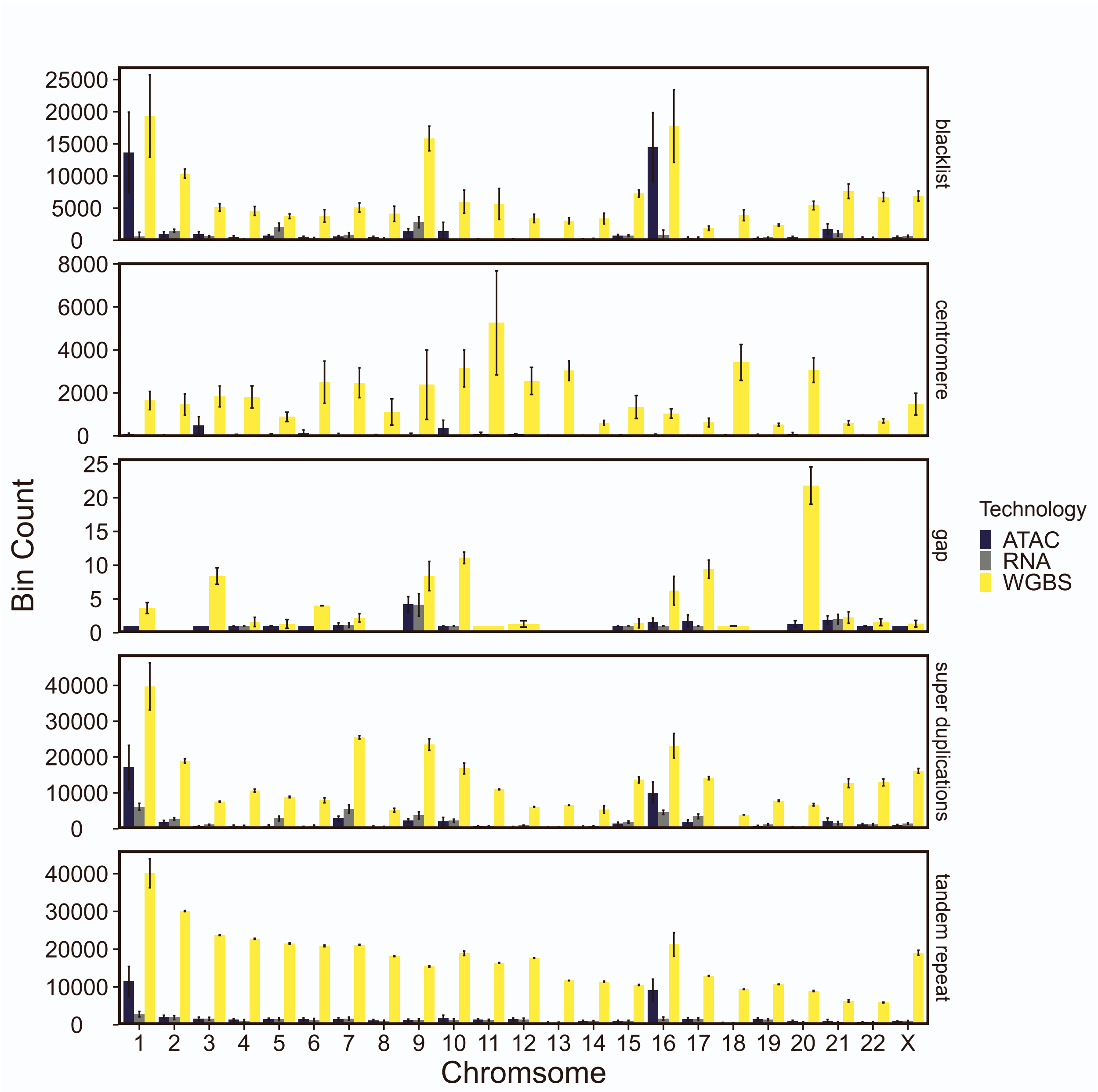

Supplemental Figure 2: <u>Count of Bins Intersecting hg38 SV Annotations, related to Figure 4.</u> This figure visualizes the mean number of bins (equal and unequal length) that intersect hg38 blacklist, centromere, gap, large duplication, and tandem repeat annotations. Data are separated by technology and plotted as the mean and standard deviation between individuals, parents, and replicates.

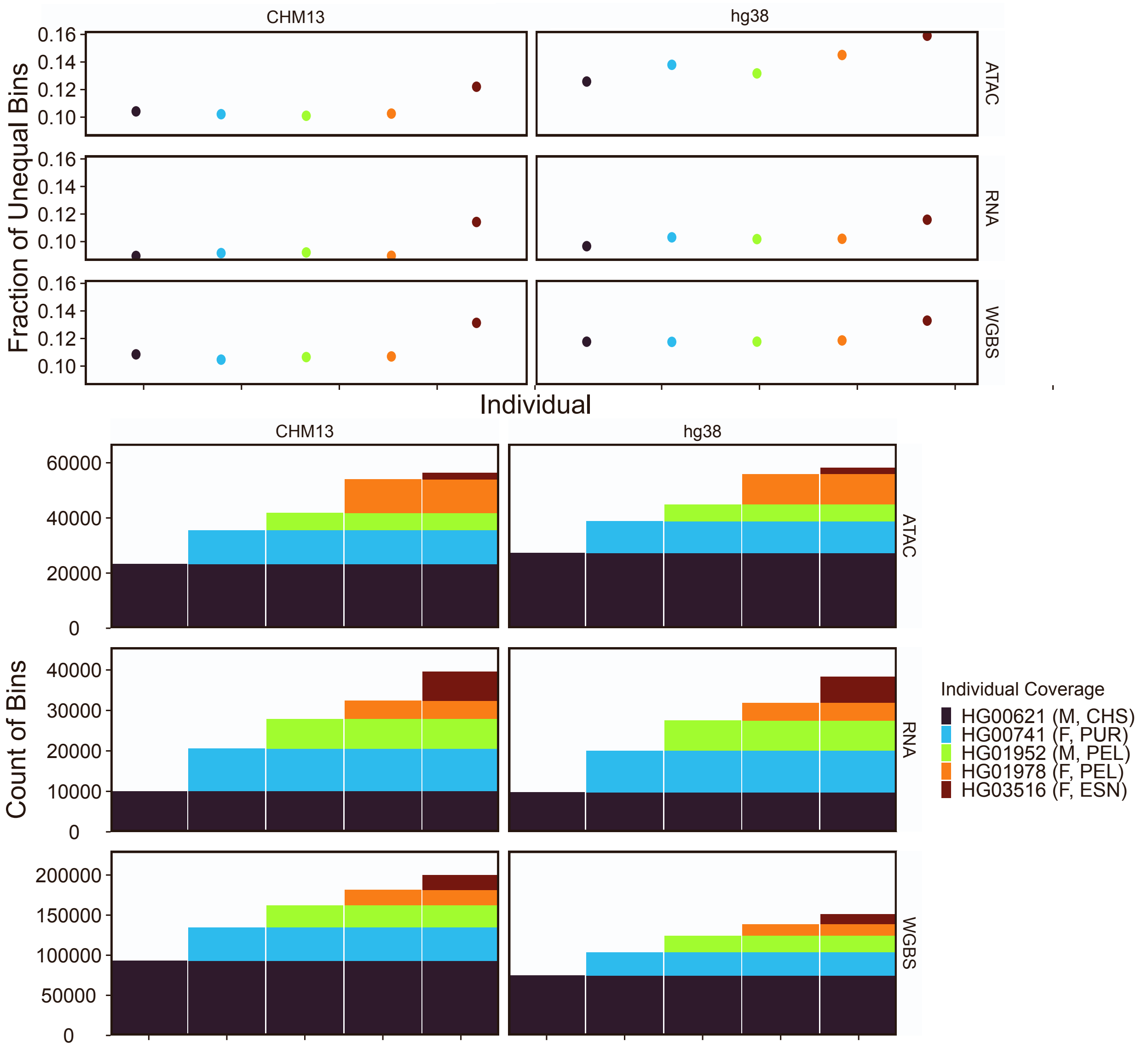

Supplemental Figure 3: <u>Bias visualized across ancestry, related to Figure 5.</u> A) The fraction of the total genome that has structural variation as measured by the fraction of unequal length bins across individuals relative to either CHM13 or hg38. B) Saturation analysis showing the cumulative number of new bins affected by reference alignment bias (RAB) as each additional individual is included, starting from HG00621. Individuals are ordered by ancestry: Han Chinese, Puerto Rican, Peruvian, and Esan.

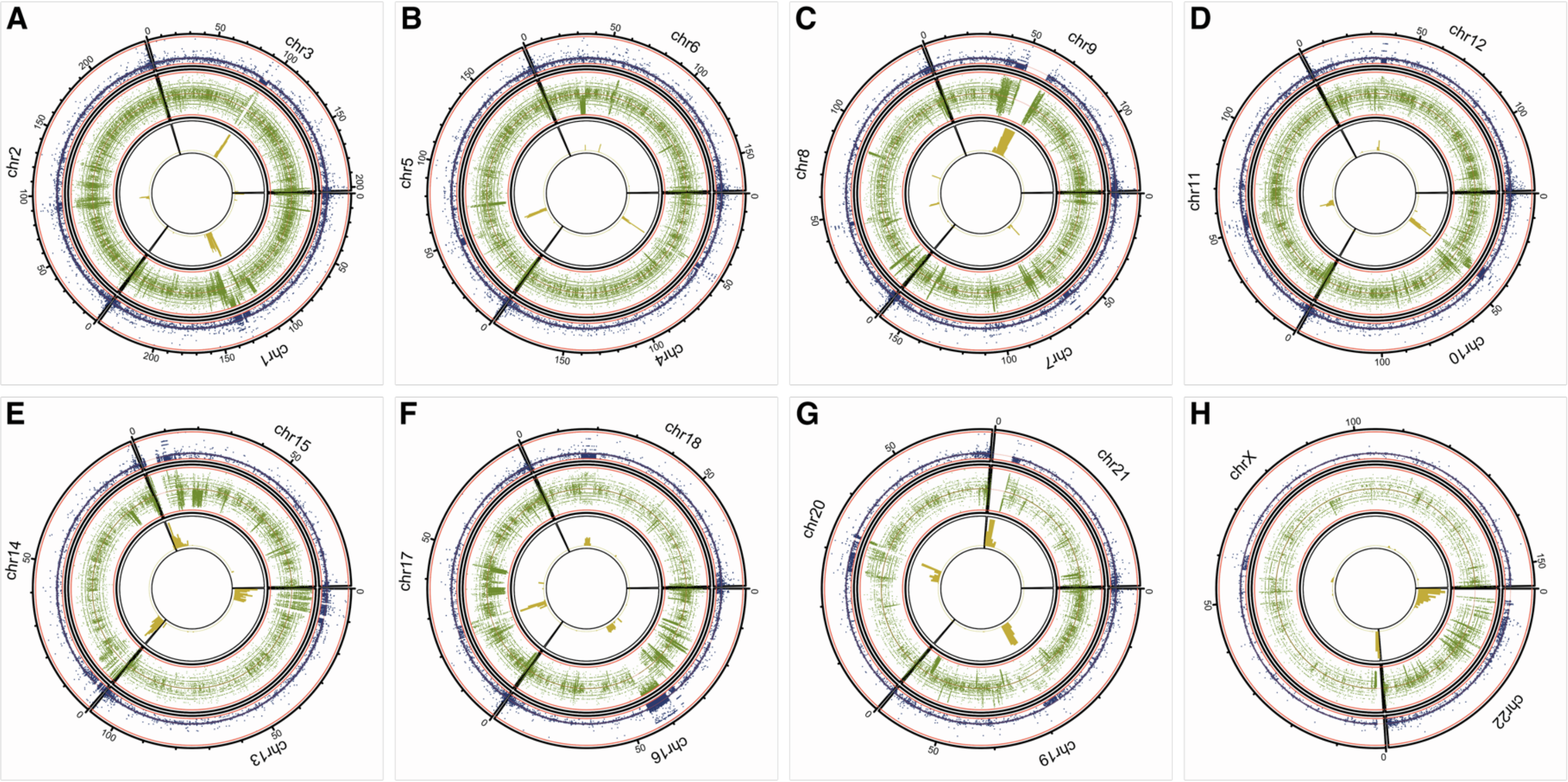

Supplemental Figure 4: Chromosome-level visualization of RNA-seq data aligned to HG01952 maternal assembly, related to Figure 6. Each chromosome is annotated by bins that did not liftover (inner track), bin reference fraction plotted for visualizations of variation from 0.5 (middle track), and bin length difference bounded from -250 to 1000 base pairs (outer track).

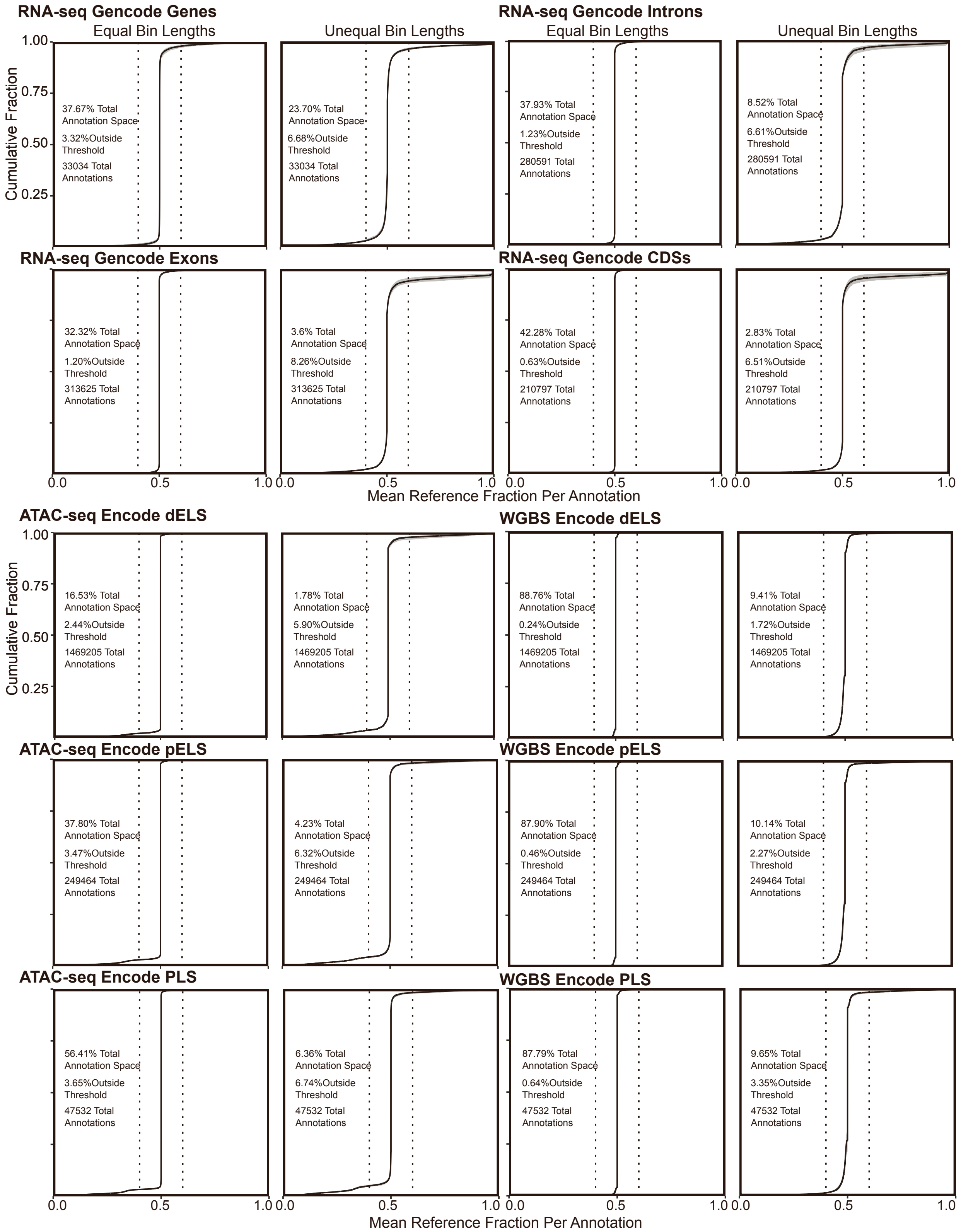

Supplemental Figure 5: Analysis of Reference Alignment Bias (RAB) Across Genome Annotations, related to Figure 4. This figure visualizes the cumulative distribution of mean reference fractions per annotation, assessing the extent of RAB within genomic data.

ATAC-seq and WGBS intersected bins were mapped to ENCODE promoter (PLS), distal enhancer (dELS), and proximal enhancer (pELS) annotations. RNA-seq intersected bins were mapped to GENCODE CDS, UTR, intron, and gene annotations. Data are split between annotations intersected with equal and unequal bin length Rf.

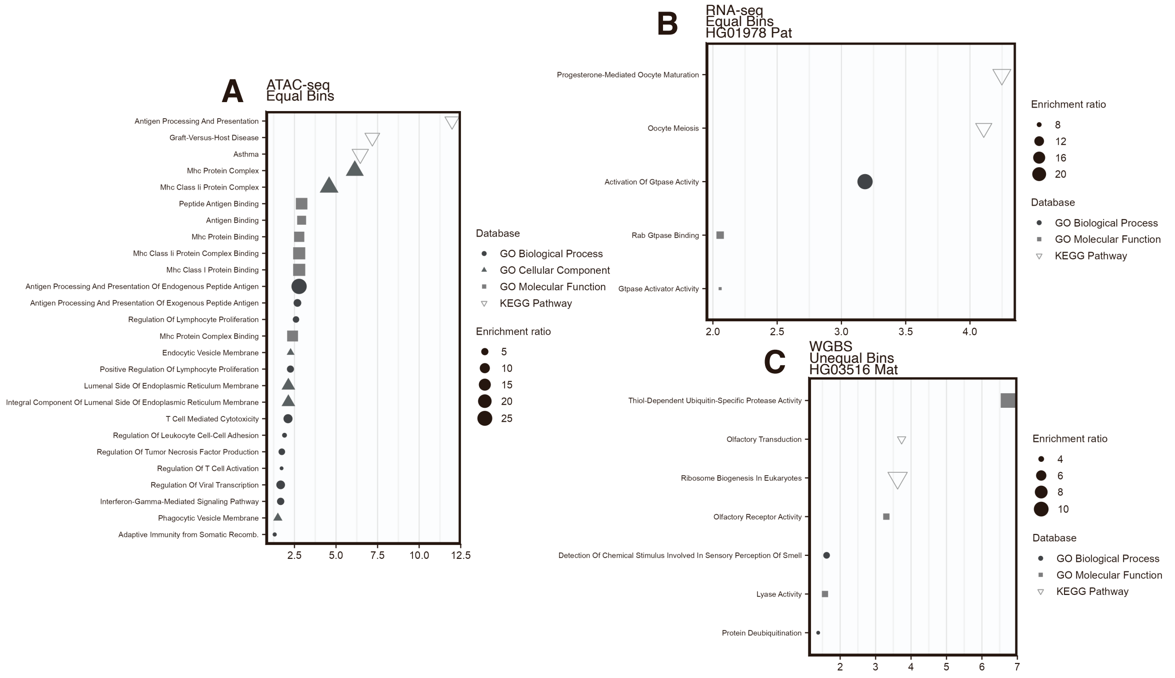

Supplemental Figure 6: <u>Representative over-representation analysis (ORA) plots across various strata, related to Figure 4.</u> x-axis: -log10(FDR). y-axis: top annotations per database with significance (FDR) < 0.05. Point size represents enrichment ratio, and point shape represents the source database of the annotation. **A)** ORA for all genes with ATAC-seq signal in equal-length bins. Most results are related to immune function, consistent with previous findings that show high genetic diversity in these genes. **B)** ORA for all genes with RNA-seq signal from the paternal haplotype of individual HG01978 falling into equal-length bins. **c:** ORA for all genes with whole genome bisulfite sequencing (WGBS) signal from the maternal haplotype of individual HG03516 falling into unequal length bins.

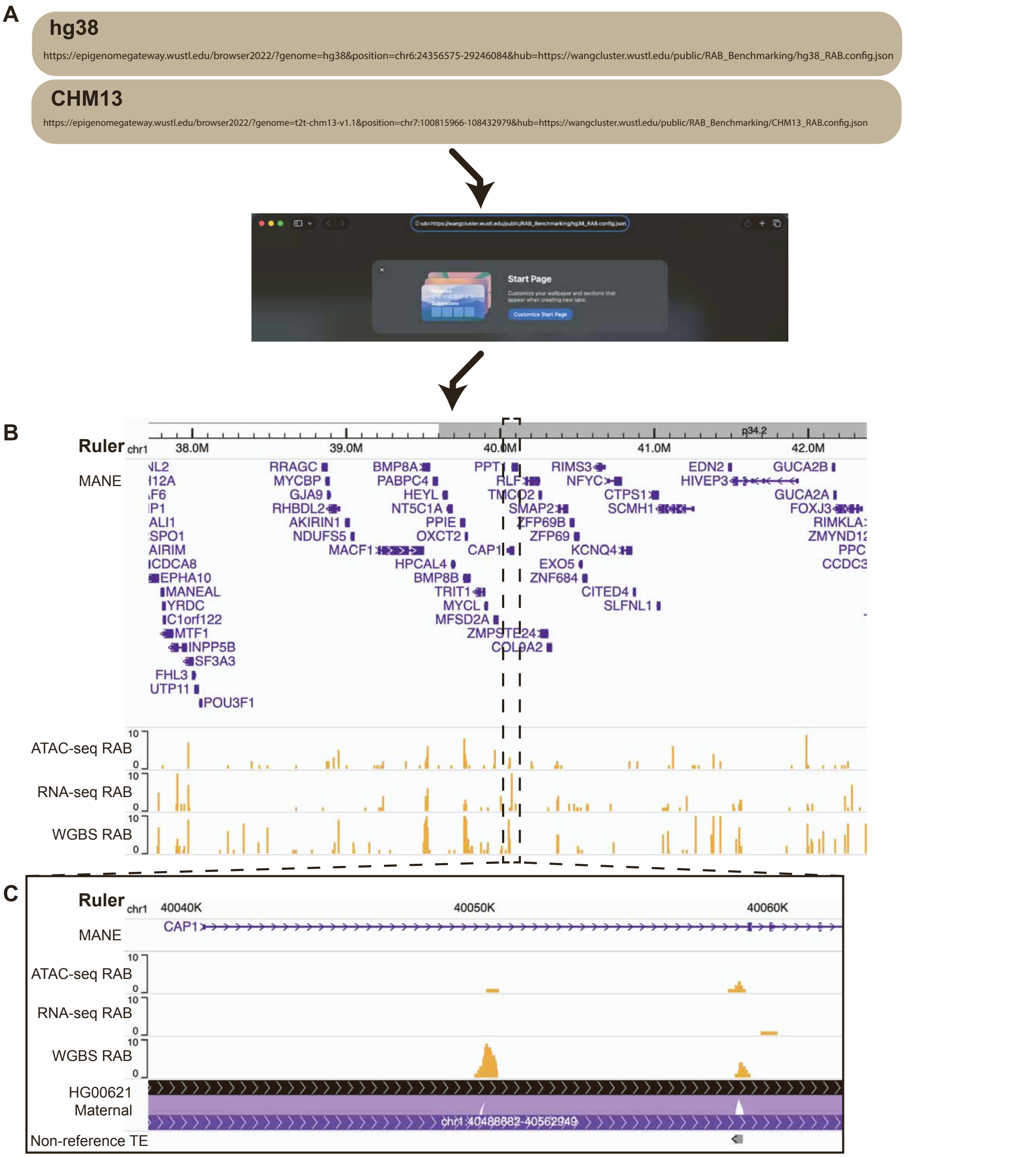

Supplemental Figure 7: Example of genome-wide RAB annotations and instructions for browser access, related to Figure 6. A) Instructions for accessing the genome-wide RAB annotations on the WashU Epigenome Browser. To view the tracks, click the provided link or paste the URL into a web browser. The hg38 annotations can be accessed at https://epigenomegateway.wustl.edu/browser2022/?genome=hg38&hub=https://wangcluster.wustl.edu/public/RAB_Benchmarking/hg38_RAB.config.json, and the CHM13 annotations at https://epigenomegateway.wustl.edu/browser2022/?genome=t2t-chm13-v1.1&hub=https://wangcluster.wustl.edu/public/RAB_Benchmarking/CHM13_RAB.confi

g.json. B) Example of a genomic region on chromosome 1 annotated for recurrent RAB across assays. C) Detailed view within this region showing the CAP1 gene, where two non-reference TE insertions are present, including a partial insertion found in a subset of samples and a complete Alu element, illustrating how sequence differences relative to the reference can contribute to local reference alignment bias.
