# Supplementary material for: Quantifying reference alignment bias in functional genomics analyses
Source: Cell Rep Methods. 2026 May 14;6(7):101461. doi: 10.1016/j.crmeth.2026.101461 (PMC13390086; doi:10.1016/j.crmeth.2026.101461)
Supplement: Document S2. Article plus supplemental information [file mmc7.pdf]

Cell Reports
Methods

Resource

Quantifying reference alignment bias in functional genomics analyses

Graphical abstract

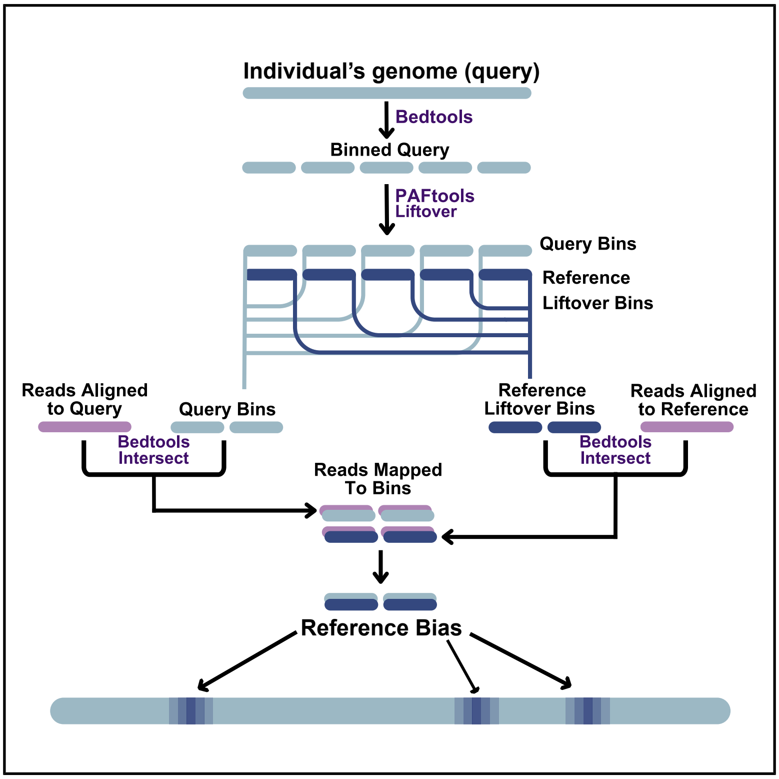

Authors

Nina Tekkey, John E. Garza, Wenjin Zhang, ..., Xiaoyun Xing, Juan F. Macias-Velasco, Ting Wang

Correspondence

juanfmacias@wustl.edu (J.F.M.-V.), twang@wustl.edu (T.W.)

In brief

Tekkey et al. present a workflow for quantifying the bias introduced into functional genomics assays from reference alignment. By comparing read alignments to *de novo* genome assemblies and human reference genomes, they reveal the amount and location of bias contributable to the use of a reference genome.

Highlights

- We provide a method for quantifying reference alignment bias in a genome-wide manner
- 0.2%–3% of the genome is susceptible to bias when using human reference hg38
- Bias persists even with the use of the complete human reference CHM13
- Genome-wide quantification highlights problematic regions of RAB

Tekkey et al., 2026, Cell Reports Methods *6*, 101461
July 20, 2026 © 2026 The Author(s). Published by Elsevier Inc.
https://doi.org/10.1016/j.crmeth.2026.101461

CellPress

Cell Reports Methods

CellPress
OPEN ACCESS

Resource

Quantifying reference alignment bias in functional genomics analyses

Nina Tekkey,[1] John E. Garza,[2] Wenjin Zhang,[1] Derek Albracht,[2] Chad Tomlinson,[2] Edward A. Belter,[2] Xiaoyun Xing,[1] Juan F. Macias-Velasco,[1,2,*] and Ting Wang[1,2,3,*]
[1]Department of Genetics, Washington University School of Medicine, 660 South Euclid Ave., Saint Louis, MO, USA
[2]McDonnell Genome Institute, Washington University School of Medicine, Saint Louis, MO, USA
[3]Lead contact
*Correspondence: juanfmacias@wustl.edu (J.F.M.-V.), twang@wustl.edu (T.W.)
https://doi.org/10.1016/j.crmeth.2026.101461

MOTIVATION Probing genomic function (chromatin accessibility, expression, and DNA methylation) is typically done through next-generation sequencing (NGS) assays, which rely on alignment to linear haplotype reference genomes. Alignment to a reference genome can result in a biased interpretation of NGS data through systemic errors known as reference alignment bias (RAB). The downstream consequences of RAB on alignment are an over- or underestimation of function; however, the degree to which RAB impacts functional genomics readouts has not been thoroughly quantified. We therefore sought to develop a framework to systematically quantify the extent and genomic distribution of reference alignment bias in functional genomics assays, providing insights and recommendations for the use of reference genomes in analyzing these data.

SUMMARY

When aligning next-generation sequencing (NGS) reads to a reference genome, differences between the true genome of the individual under study and the reference result in a biased interpretation of aligned data through systematic errors known as reference alignment bias (RAB). The degree to which RAB impacts functional readouts has not been thoroughly quantified. Leveraging resources from the Human Pangenome Reference Consortium, here we quantify RAB in functional genomics assays. Our results indicate that, on average, 0.2% of the genome is susceptible to bias in RNA sequencing (RNA-seq) studies, 1% in ATAC-seq, and 3% in WGBS when using the human reference hg38. Our study quantifies the effect of RAB on functional assays and highlights the importance of using an adequately representative reference genome.

INTRODUCTION

Understanding genomic function is critical to advancing our knowledge of all domains of human biology. Probing genomic function (chromatin accessibility, expression, and DNA methylation) is typically done through next-generation sequencing (NGS) assays.[1–4] These assays return "reads," which are partial sequences of an individual's genome that, when aligned to a fully assembled genome, inform us of the functional landscape of the genomic regions to which they are aligned. Current NGS workflows rely on one common step: alignment of reads to a reference genome.

The current human reference is Genome Reference Consortium Human reference 38, or GRCh38 (referred to as hg38 from here on).[5] The current structure of hg38 is that of a linear, mosaic haplotype, predominantly derived from a single individual, with fragments from over 50 others mixed in.[6] Despite its status as the standard, hg38 harbors inaccuracies, encompassing errors, rare structural variants, and genomic gaps in regions too difficult to assemble correctly.[7] The telomere-to-telomere reference, T2T-CHM13, represents an improvement on hg38, resolving gaps in centromeric and chromosome short-arm regions.[8] Even so, the linear structure of both hg38 and CHM13 fundamentally collapses the human genome's variability into a single sequence. This condensation not only underrepresents human genetic variation, leading to bias in variant detection, but also seeds unquantified systemic errors into functional genomic analyses.

Differences between an individual's true diploid genome and the reference used during alignment lead to what is known as reference alignment bias (RAB). These biases manifest as systemic errors, skewing the interpretation of NGS data and potentially misguiding subsequent biological insights. Here, we seek to provide a new measure of RAB to inform future analyses and provide a population-scale view of where bias appears in the genome, aiding future development of algorithms and software tools for bias correction.

© 2026 The Author(s). Published by Elsevier Inc.
This is an open access article under the CC BY license (http://creativecommons.org/licenses/by/4.0/).

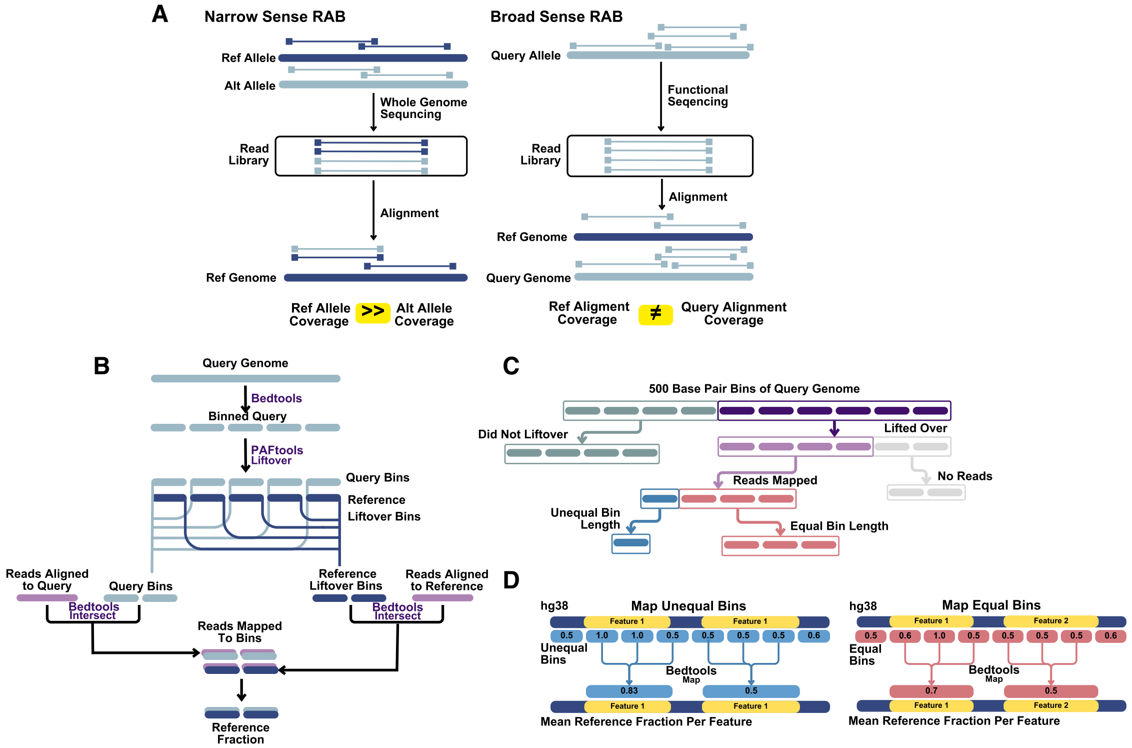

Figure 1. Overview of RAB quantification

(A) Narrow-sense RAB describes the decreased mapping of reads spanning an alternative allele compared to reads spanning a reference allele when aligned to a reference genome. This results in an allele frequency ratio that differs from the real ratio of 1:1. Broad-sense RAB describes the difference in mapping of reads spanning any given query allele when those reads are aligned to a reference versus the original genomic sequence.
(B) Workflow for quantifying the difference in aligned reads for syntenic regions of the individual and reference genome.
(C) Bin fate categorization: This represents the possible outcomes for bins post-liftover, indicating the variation present in their originating regions.
(D) Associating Rf with genomic annotations: Bias quantification in relation to genomic annotations is achieved by mapping the Rf of bins overlapping annotations by at least 51%.

Traditional RAB metrics center on allelic frequency, variant detection, and genome-wide association study (GWAS) outcomes.[9–11] Recent studies show that graph genome alignments of ChIP-seq and ATAC-seq data from monocyte-derived macrophages reveal an additional 2%–3% of peaks versus linear reference.[12] These results support the conclusion that linear genomes contribute to RAB, but they do not provide insight into how RAB appears in alignments or how much RAB one can expect when aligning to a reference genome. In the context of variant detection, RAB can be observed in alignments over heterozygous sites, within which one allele is the hg38 sequence and the other is an alternative allele. Genome-wide, at these sites, the hg38 allele will have disproportionately higher coverage. Quantifying this shift is one way in which RAB has been estimated.[10] This metric encapsulates the tendency of reads to map more effectively and, as a result, more abundantly when the sequence of the read is closer to that of the reference. When two alternative alleles exist at a given locus, alleles closer in sequence to hg38 will have higher coverage on average.[3,10,11] This phenomenon is a narrow sense of RAB, in which bias is quantified as reads mapping or failing to map to a specific locus due to an individual's local variants, leading to a deviation from the expected allelic ratio of 1:1 (Figure 1A). This approach works because coverage differences at heterozygous sites are solely attributable to alignment bias, under the assumption of equal representation.[10] Here, we refer to this as the narrow sense because it captures only the direct and local effect of sequence similarity on alignment, in a manner analogous to how narrow-sense heritability captures only additive genetic effects.[13] In both cases, the measure reflects a simple and intuitive mechanism but does not encompass the broader set of influences that can exist. Just as broad-sense heritability accounts for additional sources of genetic variance beyond additivity,[14] RAB can also arise from genome-wide variation that influences read alignment even at distant loci.

Current tools to address RAB, such as ReferenceFlow and Biastools, focus primarily on narrow-sense bias, quantifying deviations in allelic balance at heterozygous sites using whole-genome sequencing data.[11,15] These approaches operate within variant-based frameworks that require a predefined set of variants identified through alignment to a reference genome. Because variant discovery itself is influenced by RAB, the sensitivity and accuracy of these tools are inherently constrained. As a result, they can only assess bias in regions where variation has already been reliably detected, leaving structurally complex or challenging regions an outstanding challenge. Moreover, these

methods are rooted in assumptions appropriate for variant calling but not for functional genomics. In particular, they rely on the expectation of a 1:1 coverage ratio between alleles, an assumption that does not hold when assays measure activity rather than genotype.

In functional genomics, differences in expression, accessibility, or methylation between alleles can produce unequal coverage independent of alignment bias. Furthermore, narrow-sense approaches treat each allele as independent and do not account for how the global structure and sequence context of an individual's genome can influence alignment outcomes at specific sites. To address these limitations, we explore a broader sense of RAB, in which bias arises not only from local variation but also from the total sequence and structural divergence between an individual and the reference.

At the level of read alignment, broad-sense RAB manifests as altered read fate (mis-mapping, failing to map, or multi-mapping) when aligning to a reference genome divergent from the true genome. These shifts in read placement distort coverage profiles and, in turn, our interpretation of genomic function.[3,4] We define broad-sense RAB as the deviation in read placement or coverage as a function of the holistic sequence and structural differences between the reference and individual genomes, including variants and configurations that cannot be fully captured through conventional variant-calling approaches. By leveraging high-quality, haplotype-resolved assemblies across multiple individuals, we aim to quantify this broader bias and reveal how it manifests across populations.

Studying broad-sense RAB requires highly complete, accurately phased assemblies from individuals for whom functional genomics data can also be obtained. The Human Pangenome Reference Consortium (HPRC) was established to overcome the limitations of the linear reference genome and to reduce RAB[7,16] by adopting a graph-based genomic structure that captures a broader spectrum of human genetic variation.[17] Although this framework is expected to mitigate bias, it has not yet been widely adopted for functional genomics. As an immediate benefit of the HPRC effort, the availability of high-quality, haplotype-resolved assemblies and matched cell lines provides a unique opportunity to begin profiling broad-sense RAB relative to hg38 and CHM13. Our objective is to systematically quantify and contextualize RAB within functional genomics analyses to pinpoint where and how bias manifests. Such an approach will make it possible to identify genomic intervals that are prone to broad-sense RAB and to highlight where more representative approaches or long-read methods may be needed to achieve accurate interpretation.

In this study, we employed data from immortalized lymphoblastoid cell lines (LCLs) from the 1000 Genomes Project,[18,19] which were subsequently utilized by the HPRC for generating comprehensive genomic data. Specifically, we leverage functional genomics data from individuals HG01952, HG00741, HG00621, HG01978, and HG03516. Utilizing resources for these samples, this study aims to quantitatively assess RAB across three functional genomics assays (RNA-seq, ATAC-seq, and WGBS) employing respective aligners. Through a comparative analysis of alignment coverage to individual-specific, hg38, and CHM13 references, we quantify and contextualize RAB in functional genomics.

Our approach employs phased *de novo* genome assemblies from these five individuals, paralleled by the analysis of their corresponding NGS datasets across the mentioned functional assays (Figure 1). For each dataset, we compare the alignment of NGS reads against a reference to the alignment of the same reads against the respective individual's actual genome. This comparison serves as the core of our investigation into RAB. Utilizing these data, we quantify the magnitude of RAB imparted on each genomic technology. This quantification is conducted at multiple scales, including genome-wide, chromosome-wide, and feature-specific levels, enabling a comprehensive understanding of read placement alterations attributable to RAB. This work establishes the framework and initial dataset for a population-aware catalog of RAB. Future expansions of this catalog will enable users to assess the reliability of their analyses and identify when a chosen reference may introduce bias.

RESULTS

Read phasing generates a truth set for each parental haplotype

NGS data in this study had to first be phased by parental origin to avoid cross-parental artifacts. Phasing was accomplished by attributing reads to one or both parental genomes using a mapping quality (MAPQ) score from alignments to the individual and combined parental assemblies. This method is limited by the nature of MAPQ scores, which are a measure of both mappability and read quality.[20] In contrast to other read phasing tools such as WhatsHap, this method does not rely on a reference genome and, unlike WhatsHap, can account for sizable structural variation, both of which are vital for the purpose of this study.[21] Figure 2 displays the composition of phased reads attributed to maternal, paternal, or both genomes, segmented by assay type, individual, and replicate. Across all samples, the overwhelming majority of reads were assigned to both parental genomes (ATAC-seq: 79.2% ± 10.85, RNA-seq: 91.3% ± 1.05, WGBS: 88.8% ± 1.27). A consistently higher proportion of reads was allocated to the maternal genome, consistent with the inclusion of the mitochondrial genome in the maternal assembly and, to a lesser extent, with the larger size of the X chromosome relative to the Y chromosome. Variability in the proportion of maternal reads among ATAC-seq datasets was observed, attributable to differing levels of mitochondrial contamination across individuals, thus elevating the share of reads ascribed to the maternal genome. Any incorrectly phased reads would violate our assumption that all reads aligned to the parental genome are derived from that genome, possibly resulting in an overestimation of bias.

Splitting the genome into bins provides a genome-wide view of variation

To evaluate RAB across the entire genome in a consistent and comparable way, each query genome was divided into 500 bp bins spanning its full length. These bins were lifted to hg38 to generate syntenic bin pairs, each representing a query interval

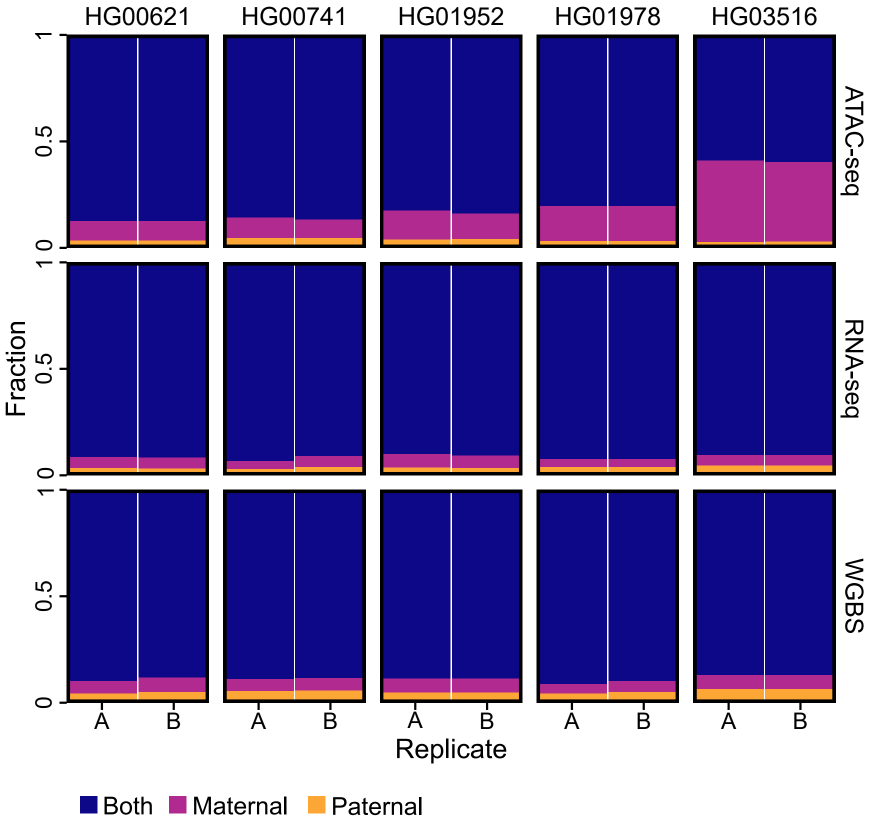

Figure 2. Distribution of phased read attribution across technologies and samples

This figure illustrates the proportion of reads attributed to maternal, paternal, or both genomes for each sequencing technology (ATAC-seq, RNA-seq, and WGBS) across all individual samples and replicates.

and its corresponding reference interval (Figure 1B). Bin pairs were then categorized according to their post-liftover outcomes, which included equal length, unequal length, or failed liftover. Successful liftovers indicate sufficient synteny to define corresponding intervals, with differences in length reflecting structural variation between the query and reference genomes. Failed liftovers represent intervals where synteny could not be established, corresponding to regions absent or too structurally complex to be represented in the reference (Figure 1C). In addition to these categories, syntenic bin pairs in which both the query and reference alignments contained fewer than ten mapped reads were excluded from RAB quantification, as coverage was insufficient to reliably assess bias.

Distributions of bin pair outcomes and coverage across each query genome reveal clear differences among functional genomics assays (Figure 3; Table S1). On average across all samples, approximately 15 percent of syntenic bins in RNA sequencing (RNA-seq) and ATAC-seq and 98 percent in WGBS contained at least 10 mapped reads (Table S1). These differences reflect the distinct genomic scopes of the assays, with WGBS providing near-complete genome-wide coverage, while ATAC-seq and RNA-seq target accessible chromatin and expressed regions, respectively. Across all technologies, syntenic bin pairs of equal lengths notably outnumber those with unequal lengths (approximately 8, 5, and 7 times as many, respectively), consistent with the expected relative rarity of structural variants that induce size differences.

Genome-wide and chromosomal-level RAB

To quantify broad-sense RAB, we compared read coverage between each pair of syntenic bins derived from the query and reference genomes. For each pair, we calculated a reference fraction (Rf), defined as the number of reads aligning to the reference genome divided by the sum of reads aligning to both the individual's and the reference genomes (Figure 1B; Equation 2). This metric provides a per-bin measure of alignment bias, where an unbiased interval has an Rf of 0.5, indicating equal representation between the two alignments. Values greater than 0.5 indicate more reads aligning to the reference genome, whereas values below 0.5 indicate a bias toward the individual's genome. To evaluate the extent and variability of this bias, Rf values were summarized both genome-wide and for each chromosome.

Figure 4 presents the cumulative distribution of Rfs for all syntenic bin pairs, analyzed genome-wide (Table S1). Each curve shows the mean Rf across individuals, with the shaded region indicating the range across samples. The portion of the genome analyzed is expressed as the percentage of all query bins that could be lifted over to the reference, had sufficient coverage, and were classified as equal- or unequal-length pairs. Bins with Rf values outside the 0.4–0.6 range were classified as affected by RAB, where Rf > 0.6 indicates a bias toward the reference and Rf < 0.4 indicates a bias toward the individual. An Rf of 0.6 corresponds to an overestimation using the reference of more than 50 percent relative to the reference estimate, while an Rf of 0.4 corresponds to an underestimation of about 33 percent. The proportion of bins exceeding these thresholds, scaled by the fraction of the genome assayed, estimates the total genomic space affected by RAB.

For ATAC-seq, approximately 6.93% of bins were of equal length and 1.14% were of unequal lengths. Among bins of equal length, 8.36% showed evidence of RAB, compared to 35.49% of bins with unequal lengths. Together, this indicates that for ATAC-seq alignments to hg38, about 0.983% of the genome exhibits measurable bias, with 0.579% arising from equal-length bins and 0.404% from unequal-length bins. This corresponds to roughly 12% of all bins with read coverage in ATAC-seq, reflecting that a modest but nontrivial fraction of the assay's target space is affected by alignment bias.

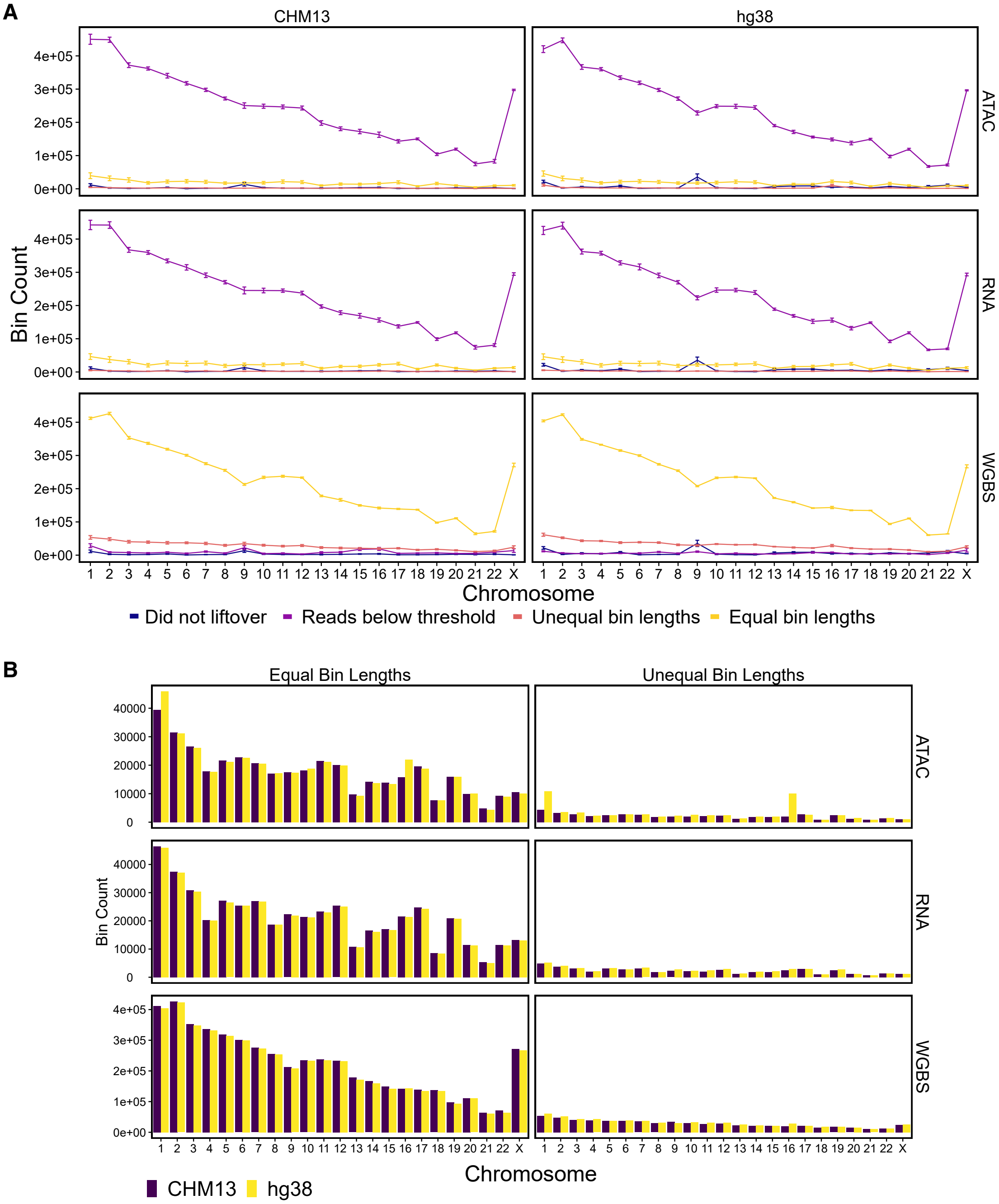

Figure 3. Distribution of bin fates across chromosomes by sequencing technology and reference

(A) Visualizing the composition of bin fates across all chromosomes, analyzed separately for each of the three main functional genomics assays: ATAC-seq, RNA-seq, and WGBS, and plotted as the mean and standard deviation across individuals and replicates. The *y* axis denotes the "bin count," representing the number of bins within each category of fate. Bins with equal lengths (yellow) indicate regions without structural variation.

(B) Total count of bins analyzed in downstream analysis. This figure visualizes the total bins retained for further downstream analysis (those with 10 or more intersecting reads), plotted as the mean across individuals and replicates.

For RNA-seq, about 8.00% of bins were of equal length, with 1.34% affected by RAB, and 0.94% were of unequal length, of which 11.00% were affected by RAB. Together, this indicates that approximately 0.210% of the genome is affected by RAB in RNA-seq alignments to hg38, with 0.107% arising from equal-length bins and 0.103% from unequal-length bins. This corresponds to about 2.4% of all bins with read coverage in RNA-seq, reflecting both the restricted genomic scope of the assay and the comparatively lower sequence variation within exonic regions.

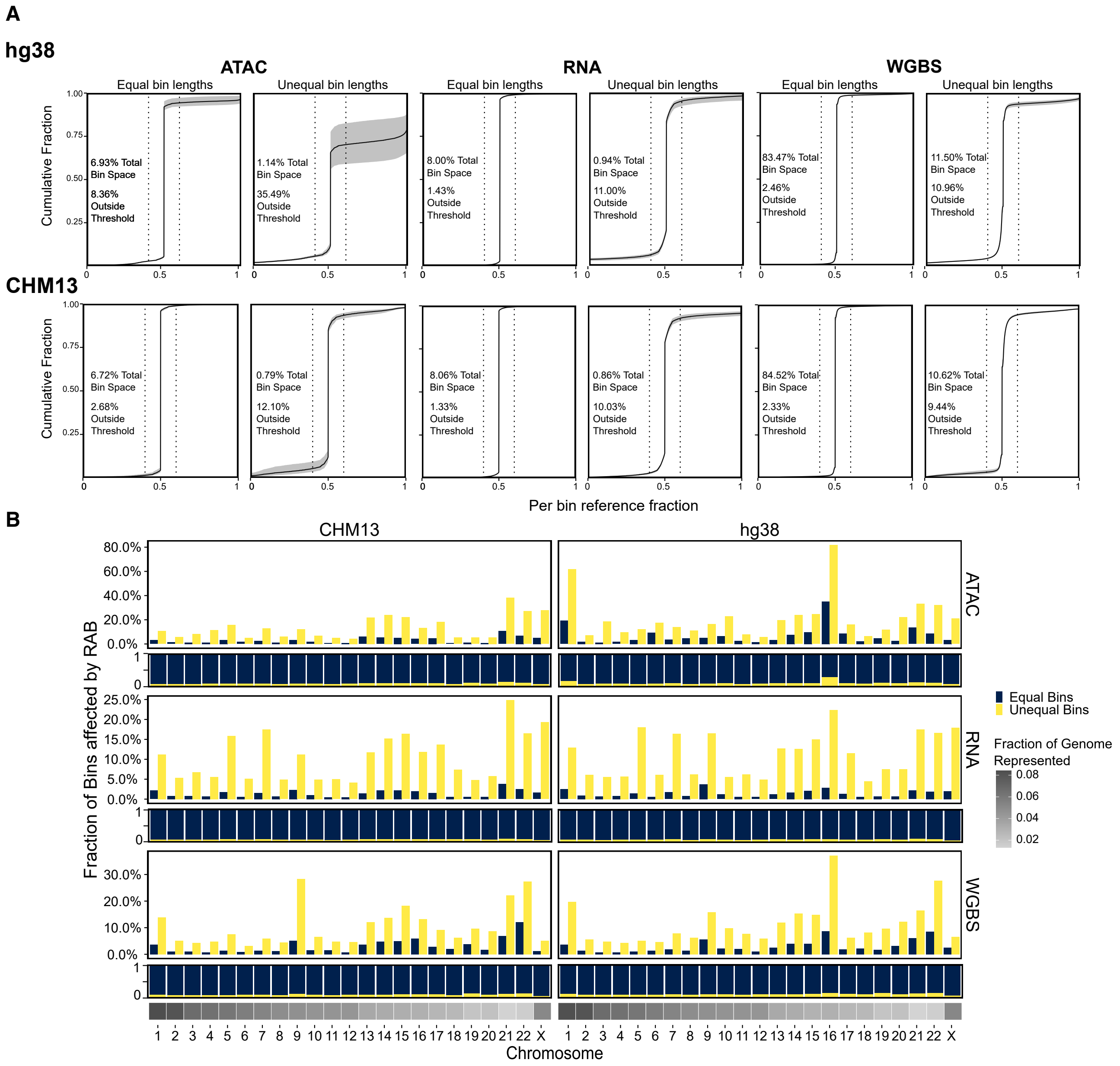

Figure 4. Analysis of RAB across the whole genome and chromosomal levels

This figure visualizes the cumulative distribution of bin reference fractions, assessing the extent of reference alignment bias (RAB) within genomic data.

(A) Genome-wide distribution analysis: Panels display the genome-wide cumulative distribution curves of bin reference fractions (Rf). Each curve represents the aggregated average across all samples, with the shaded region representing the range. Curves are subset by bin outcome: equal-length bins or unequal-length bins.

(B) Chromosomal-level distribution analysis: Panels in section B further dissect the distribution of bin reference fractions by individual chromosomes for each sequencing technology. Chromosomes Y and M were excluded from this analysis. The percentage of equal- or unequal-length bins that exhibit RAB is shown at the chromosomal level, along with the fractional amount of each type of bin per chromosome and the total amount of genomic space each chromosome represents.

For WGBS, 83.47% of bins were of equal length, with 2.46% affected by RAB, and 11.50% were of unequal length, of which 10.96% were affected by RAB. Together, this indicates that approximately 3.31% of the genome is affected by RAB in WGBS alignments to hg38, with 2.05% arising from equal-length bins and 1.26% from unequal-length bins. This corresponds to about 3.5% of all bins with read coverage in WGBS, reflecting the genome-wide scope of the assay and its increased sensitivity to variation across diverse sequence contexts.

We next examined RAB at the chromosomal level to assess its extent and variability between chromosomes. Certain chromosomes are known to be more challenging for alignment due to

their sequence composition and structural complexity, and we therefore expected some to exhibit higher levels of bias. Chromosomes Y and M were omitted due to insufficient coverage for meaningful analysis. Chromosomes exhibiting the highest levels of RAB did so consistently across all three technologies, with chromosome 16 showing the strongest effect, followed by chromosomes 1 and 22. In general, there was a trend for smaller chromosomes to contain a higher proportion of bins affected by RAB (Figure 4B).

Within the unequal-length bin category in ATAC-seq, a prominent step appears in the cumulative distribution at Rf = 1 (Figure 4A; Figure S1). This reflects a large number of bins where reads map only to the reference and not to the individual's genome. Such bins typically correspond to deletions in the individual relative to the reference, which can still be lifted over and therefore remain in the analysis. In contrast, bins with Rf = 0, where reads map only to the individual and not to the reference, are comparatively rare because these sites are often insertions that fail to lift over and are thus excluded from the analysis.

Because CHM13 represents a more complete and contiguous linear reference than hg38, we next assessed whether its improved representation of repetitive and structurally complex regions reduces alignment bias. Because CHM13 represents a different sequence space, a different set of bins was used in this downstream analysis (Figure 3B). Since downstream analysis at the chromosome level is computed conditioned on this different set of bins, we cannot make direct quantitative comparisons between CHM13 and hg38 but instead qualitatively describe their differences and similarities. Using CHM13 as the reference assembly, we observed a bias of 0.27%, 0.19%, and 2.97% of the genome affected for ATAC-seq, RNA-seq, and WGBS, respectively. These values correspond to 3.6%, 2.2%, and 3.1% of bins with sufficient coverage in each assay. Bias was also visualized at the chromosomal level (Figure 4B), where chromosomes that showed the strongest bias against hg38, such as chromosomes 1 and 16, display fewer unequal-length bins in CHM13, reflecting better representation of genomic structures. These differences largely reflect structural variation that is corrected or properly represented in CHM13. In ATAC-seq, for instance, chromosomes 1 and 16 contain the highest density of regions overlapping hg38 blacklist intervals, segmental duplications, and tandem repeats (Figure S2), which are sources of bias mitigated in CHM13. In contrast, some chromosomes, such as chromosome 9 and the acrocentric chromosomes (chr13, chr14, chr15, chr21, and chr22), show a bias in CHM13. This can be attributed in part to the additional repeat-rich sequence that is added to these chromosomes in CHM13.[8]

Viewed through the lens of reference completeness versus representativeness, this result indicates that much of the genome-wide bias in hg38 stems from incompleteness, meaning missing or misassembled sequence, rather than solely from the fact that any linear reference is a single slice through human diversity. Filling assembly gaps and correcting structural errors therefore decreases the number of bins experiencing RAB relative to those without RAB, even without adding new haplotypic variation. However, the residual bias that persists in CHM13 highlights the second and irreducible component of the problem, i.e., representativeness. Even a complete but singular haplotype cannot accommodate all individual genomic configurations and thus is far from being able to fully eliminate RAB on a genome-wide level. In practice, completeness alleviates bias at the scale of loci and chromosomes, while representativeness remains the limiting factor for population-level generality.

Factors associated with RAB vary with assay type

To assess the global extent of RAB and identify its associated factors, we performed a series of Kolmogorov-Smirnov (KS) tests.[22] These tests compare observed Rf distributions to a theoretical distribution under the null hypothesis of no RAB. Resultant KS statistic values were visualized using line plots,[23,24] categorized according to reference, sequencing technology, chromosome, and difference in bin lengths (Figure 5B). Subsequently, an analysis of variance (ANOVA) and variance partitioning were employed to quantify and discern trends among the samples. The ANOVA model was as follows:

$$ks \sim individual + chromosome + individual : chromosome + replicate + \Delta binlength.$$

Significant effects were observed for all variables except for replicate in RNA-seq and ATAC-seq (Figures 5A; Table S2). Specifically, the chromosome variable exhibited the largest effect size in ATAC-seq analyses, while differences in bin length most strongly influenced the effect size for WGBS. In RNA-seq analyses, these two variables tied for the largest effect size.

Though individual and individual-by-chromosome effects were significant, chromosome and bin class explained the overwhelming majority of variance in RAB (Figure 5A). This pattern suggests that much of RAB arises from genomic contexts that are recurrently prone to alignment difficulty across individuals, such as repetitive or structurally complex regions, while individual differences may reflect subtler, background-dependent effects. To explore whether these individual differences might be influenced by genetic ancestry, we next examined the relationship between RAB and population background. The five individuals analyzed represent a range of ancestries and admixed populations (Han Chinese [HG00621], Puerto Rican [HG00741], Peruvian [HG01952, HG01978], and Esan [HG03516]). The rate of unequal-length bins follows the expected trend based on ancestry, with individuals of African ancestry showing the highest rate, followed by admixed individuals (Figure S3A). While one might expect that an individual of Esan ancestry would exhibit a higher rate of RAB unique to that individual, given the higher heterozygosity observed in African ancestry populations,[16,19] this effect was only apparent for RNA-seq, where each individual contributed a distinct subset of biased bins roughly aligning with ancestry (Figure S3B). ATAC-seq and WGBS, by contrast, showed no clear ancestry-associated pattern across our limited samples. A clearer understanding of how RAB behaves within and between populations as a function of genetic ancestry will require a much larger collection of individuals.

Having characterized broad trends in RAB across assays, chromosomes, and individuals, we next inspected specific loci to illustrate how RAB manifests within local genomic contexts. To visualize chromosome-level patterns, we generated Circos

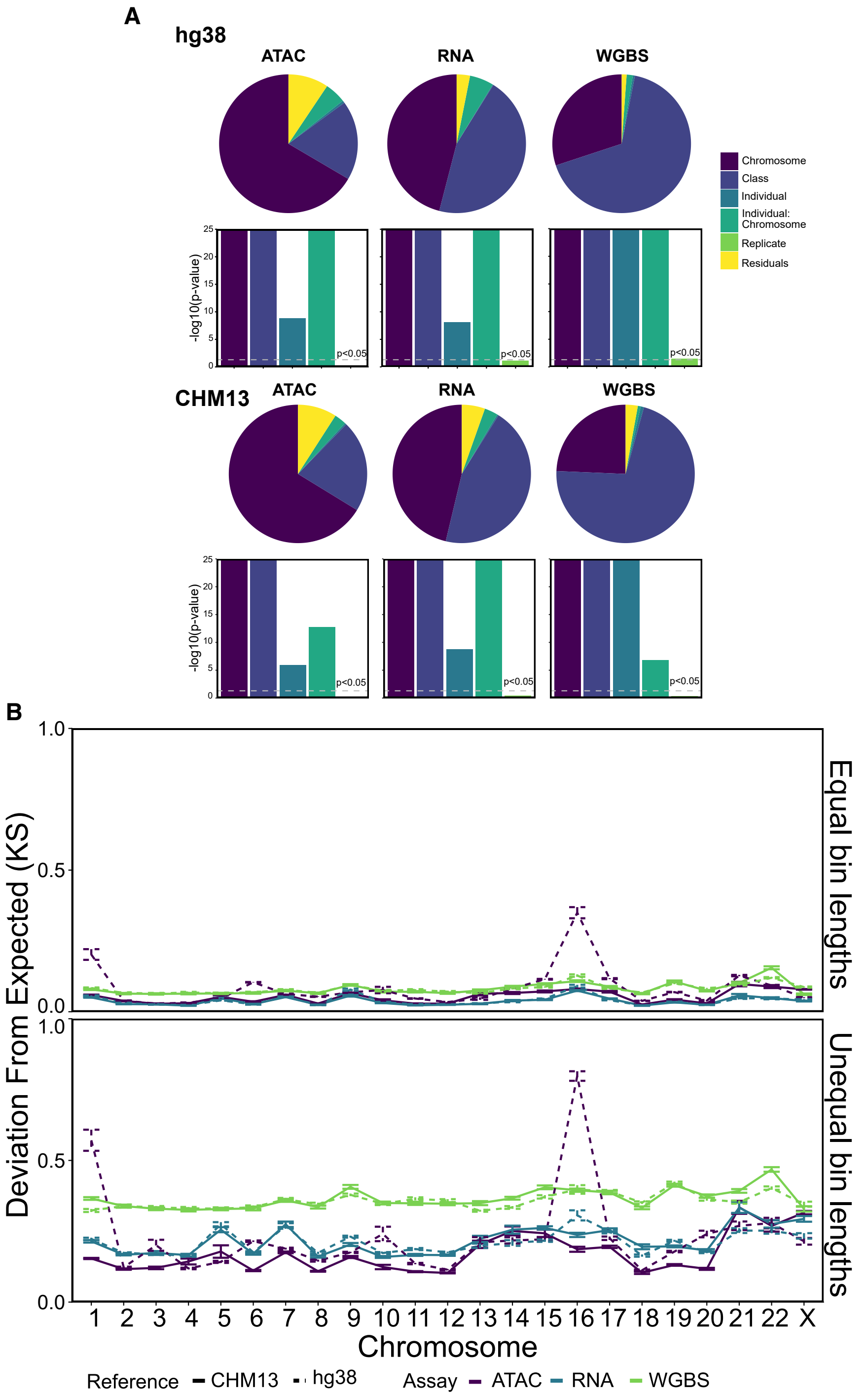

Figure 5. Statistical analysis of RAB using KS statistics

(A) ANOVA results on RAB variables: the pie charts represent the proportion of variation accountable to these variables for each technology. The represented *p* values were adjusted using the Benjamini-Hochberg FDR method.

(B) Distribution of Kolmogorov-Smirnov (KS) statistics as a function of variables: The KS statistic is plotted as the mean and standard deviation to give a consolidated view. Chromosomes Y and M have been omitted from this analysis to allow other trends to be readily observed.

plots integrating bin fate and Rf data for individual HG01952 (Figure S4).[25] This atlas highlights regions impacted by RAB, visualizing differences in Rfs, bin lengths, and the occurrence of failed liftover events. For instance, in the maternal assembly of HG01952, the Chr1p13.3 cytogenetic band is distinguished by a pronounced peak in bins where the Rf exceeds 0.5, accompanied by bin pairs of unequal lengths (Figure 6A). This region does not show the same peak in the paternal assembly of the same sample (Figure S4). Assembly alignment dot plots of this region's sequence visualize a large inversion and partial expansion in HG01952's maternal assembly relative to hg38 (Figure 6B), compared to a much smaller inversion in the paternal assembly (Figure 6C). Differences in RNA-seq coverage banding patterns between HG01952's maternal assembly and hg38, as visualized on the WashU Epigenome Browser (Figure 6D), reflect these structural differences. For this genomic region, the complexity of the maternal sequence structure is lost when aligning reads to hg38. Within chromosome 3 (Chr3p22.1) of HG01952's maternal assembly, RAB is evident at the RPSA gene locus, notably in the absence of structural variations (Figure 6E). The WashU Epigenome Browser shows a higher density of RNA-seq reads aligned to exon 2 of the maternal assembly compared to the same data aligned to hg38.[26,27] The misrepresentation of exon 2 when aligning to hg38 could then lead to downstream mischaracterization of the isoform expression of RPSA in individual HG01952.

Associating RAB with functional annotations

To assess how RAB behaves within functional annotations, bin-level Rf values were mapped to genic and putative regulatory annotations (Figure 1D; Table S3). For RNA-seq, bins were intersected with Gencode v29 CDS, UTR, intron, exon, and gene annotations.[29] For ATAC-seq and WGBS, bins were mapped to Encyclopedia of DNA Elements (ENCODE) putative regulatory annotations corresponding to distal enhancer-like (dELS), proximal enhancer-like (pELS), and promoter-like (PLS) elements (Figure S5).[30] Unlike the previous cumulative distributions of RAB, the split between annotations intersected by equal- and unequal-length bins is not mutually

CellPress
OPEN ACCESS

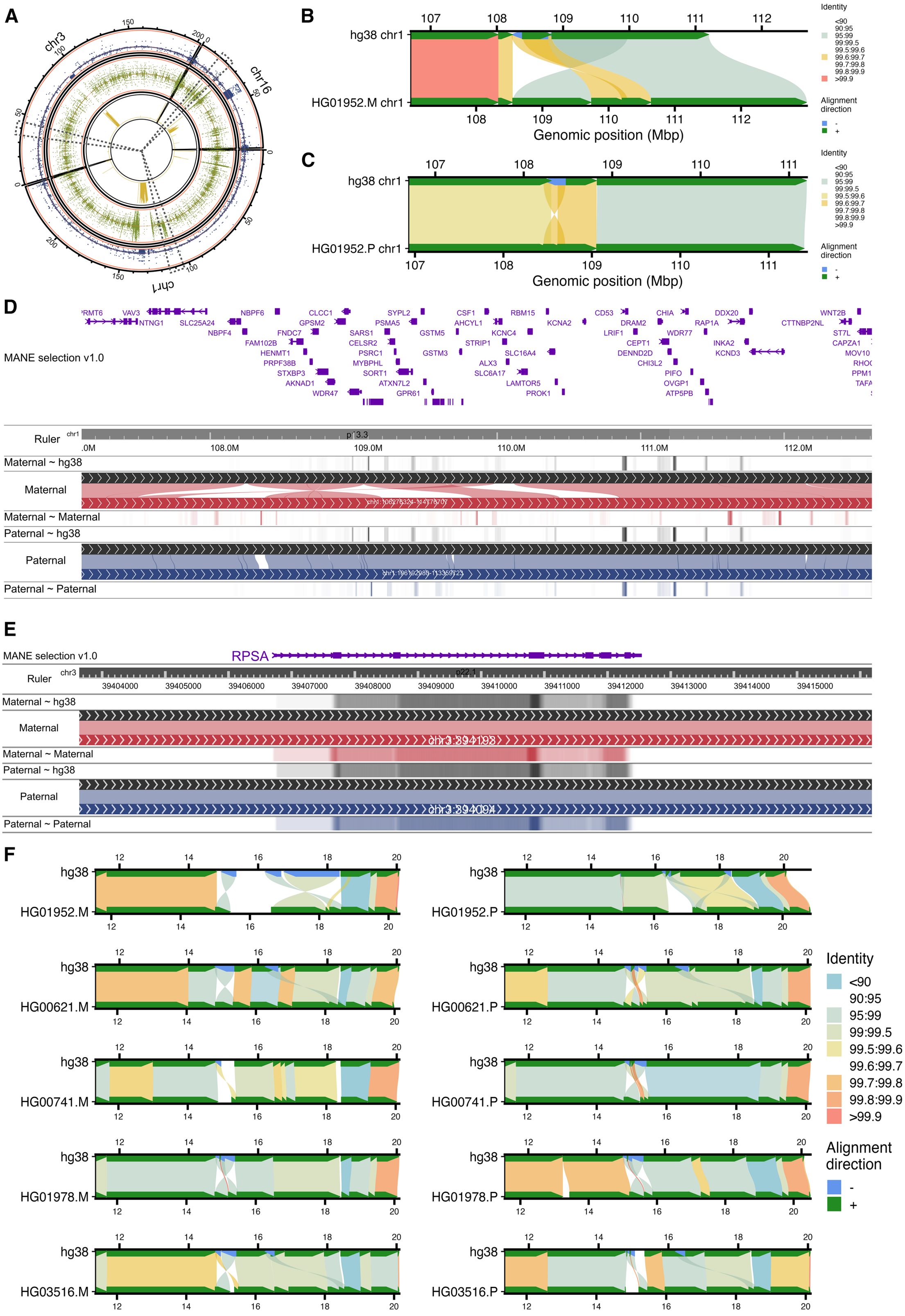

(legend on next page)

exclusive. Any given annotation may be counted twice, once in each subset of the plot, and have two corresponding mean Rf scores that relate to RAB associated with structural variation or no structural variation (Figure 1D; Equation 1).

For RNA-seq, introns were more likely to be affected by RAB than CDS when intersected with equal- or unequal-length bins (Figure S5), consistent with lower variation within coding regions.

In ATAC-seq, of all the pELS assayable, 3.47% were outside the threshold. In the unequal-length subset, 6.32% were outside the threshold. PLS elements showed a similar trend, with 3.65% outside the threshold in the equal-length subset and 6.74% outside the threshold in the unequal-length subset. For dELS, 2.55% were outside the threshold in the equal-length subset and 5.90% outside the threshold in the unequal-length subset. In each case, unequal-length intersections exhibited higher rates of RAB than equal-length intersections, consistent with their enrichment for structural differences between the individual and the reference genome.

A similar trend was observable for WGBS-intersected regulatory elements, with dELS having the least amount of bias affected and PLS the most. The percentage of total annotations analyzed for WGBS, however, is larger due to the untargeted nature of the assay (Figure S5).

Given that putative regulatory elements in our analysis frequently intersect regions affected by RAB, we next examined whether these regions correspond to particular biological pathways or functional categories by performing over-representation analyses of pathways and GO terms associated with bins affected by RAB (Table S5). ATAC-seq was the only technology for which significant results were observed when combining all individuals. Across technologies, terms related to immune response and cell signaling were most frequently associated with RAB-affected regions, consistent with their known high levels of genetic diversity (Figure S6).[31,32] Olfactory-associated terms were also enriched, in line with the extensive tandem duplication of olfactory gene families (Figure S6).[33] These data indicate that, as expected, RAB more often affects regions characterized by higher sequence diversity or structural complexity, though the magnitude of this effect varies across individuals and technologies, reflecting the targeted scope of each assay.

DISCUSSION

The advancement of NGS sequencers, aligners, and pipelines for functional genomics exploration is inherently limited by errors in alignment.[34] Thus, it is crucial to understand where biases induced by using a reference occur. Here, we have quantified RAB not as a failure of reads to align at heterozygous loci of interest but as a function of spaces in the genome. This broader-sense RAB accounts for the entirety of the alignment process and the overall representational accuracy of genomic features. It reflects how divergences between the reference and the individual's genome can lead to systematic errors in the estimation of genomic feature functions, potentially skewing our understanding of their biological roles. It is a measure of how genome-wide differences, rather than specific allelic variations, can cause shifts in read placement that affect our interpretation of genomic data on a larger scale. Our findings reveal that, on average, 0.2%, 1%, and 3% of the genome are susceptible to bias in RNA-seq, ATAC-seq, and WGBS studies, respectively, when using hg38 as a reference.

The three technologies used in this study each have unique focuses and, therefore, different implications for RAB. RAB in RNA-seq analyses can lead to downstream misrepresentation of gene expression. For ATAC-seq analyses, it may result in a failure to characterize a region as having open chromatin, and for WGBS, it may lead to mislabeling of the methylation status of single cytosines or even whole CpG islands.

All three assays were performed using identical protocols across all individuals and replicates, as the magnitude of RAB reported can be modestly influenced by assay protocol as well as data processing choices. Library construction strategies can shift which regions of the genome are effectively sampled and at what rates, which changes the relative contribution of regions that are intrinsically easier or harder to align. Additionally, read length distributions matter directly, since longer reads generally improve mappability and reduce alignment ambiguity, particularly in repetitive or otherwise challenging sequences. Therefore, the same assay performed with a different protocol on the same samples may result in a different amount of reported RAB.

Although a small percent of the genome is affected by RAB for all three technologies, the effects of this bias are not insignificant when considering the downstream consequences of misanalysis at the alignment step. For instance, the cytogenetic band Chr1p13.3 is marked by an inversion and insertion (Figure 6B) and harbors loci implicated in GWAS studies for coronary heart disease and myocardial infarction.[35] Reads from the maternal genome of HG01952 map to hg38 in a way that is inconsistent with the structure of the individual genome, which could confound the analysis of this region for disease. Consistent

Figure 6. Complex genomic variation drives RAB

(A) Chromosome-level visualization of RNA-seq data aligned to HG01952 maternal assembly.

Each chromosome is annotated by bins that did not liftover (inner track), bin reference fraction plotted to visualize variation from 0.5 (middle track), and bin length difference bounded from −250 to 1000 base pairs (outer track). Highlighted locations on chromosomes 1, 3, and 16 represent examples of RAB in the presence of structural variation at cytogenetic band Chr1p13.3, RAB in the absence of structural variation at the *RPSA* gene, and regions contributing to a significant amount of chromosome-level RAB at cytogenetic band Chr16p13.11 respectively.

(B) SVbyEye[28] alignment visualizing the alignment of the HG01952 maternal assembly to hg38 at cytogenetic band Chr1p13.3.

(C) SVbyEye alignment visualizing the alignment of the HG01952 paternal assembly to hg38 at cytogenetic band Chr1p13.3.

(D and E) (D) WashU Epigenome Browser image of cytogenetic band Chr1p13.3 and (E) WashU Epigenome Browser image of the *RPSA* gene. From top to bottom: MANE selection gene annotations, HG01952 maternal RNA-seq data aligned to hg38, HG01952 maternal assembly aligned to hg38, HG01952 maternal RNA-seq data aligned to the HG01952 maternal assembly, HG01952 paternal RNA-seq data aligned to hg38, HG01952 paternal assembly aligned to hg38, and HG01952 paternal RNA-seq data aligned to the HG01952 paternal assembly.

(F) Alignment of all 10 parental assemblies to cytogenetic band Chr16p13.11 visualized with SVbyEye.

with expectations, a greater percentage of bins impacted by structural variations showed RAB. However, when examining the overall genomic contribution to bias, bins without structural variations constituted a larger fraction of RAB-affected bins, given their much greater abundance.

The bias among bins is not independent; a bin pair with equal bin lengths may have an altered Rf because a read is aligned to a related bin elsewhere. In this way, structural variation (i.e., a change in copy number) at one locus can induce RAB at other loci. Future directions to enhance the accuracy of attributing RAB to specific types of variation could involve calculating self-similarity for every genomic bin. This would allow for the association of copy number variations with bias.

The effects of RAB are also chromosome-specific across individuals, with chromosome 16 exhibiting high amounts of RAB across all samples (Figure 6F). This effect is driven by the chromosome's enrichment in interspersed duplication blocks, with 10% of the euchromatic sequence on the short arm being composed of segmental duplications.[36] The sequence alignment of all 10 assemblies to hg38 for the cytogenetic band Chr16p13.11 shows complex and varied structural variation, which drives a significant amount of bias. The complexity of this region compared to the reference makes it difficult to attribute the reads that originate from it. Duplication of this region has been implicated as a risk factor for multiple neuropsychiatric disorders,[37] and the inability of the reference to properly represent this region poses a challenge for any attempts to probe function. Segmental duplications syntenic to this region have been previously studied in the context of primate evolution,[38] and a reconstruction of the history of this region suggests that Chr16p13.11 is the ancestral location from which segmental duplications have arisen. It may therefore be a source of structural variation.[39] In line with that possibility is the presence of a nearby putative human-accelerated region with regulatory properties.[40]

Use of the T2T reference CHM13 in place of hg38 introduces a slight improvement in RAB on a genome-wide scale for RNA-seq and WGBS but has a much larger effect on ATAC-seq. This can be attributed to the resolution of assembly faults in chromosomes 1 and 16. CHM13 is also able to resolve more structural variation, as seen in a decrease in the number of bins of unequal size (Figures 3B and 4B). Even so, most of the structural variation and bias seen when using hg38 remains, even with a resolved linear genome, and in some chromosomes, such as chromosome 9 and the acrocentric chromosomes (chr13, chr14, chr15, chr21, and chr22), there even appears to be an increase in bias (Figure 4B). This can be attributed to the same feature that seems to improve CHM13's performance: completeness. These chromosomes in particular have added sequences that range from 35 to 13 Mbp,[8] and this increase in reference space corresponds to an increase or change in bins used for our downstream analysis compared to hg38 (Figure 3B). CHM13 adds more regions of low complexity—such as the short arms of the acrocentric chromosomes or a human satellite 3 duplication in chromosome 9—and thus low mappability, constructing new sequences that can contribute to overall bias as a sink or source of incorrectly mapped reads.

Linear reference genomes cannot capture the breadth of variation that exists within the human species. This poses a challenge for accurately characterizing the biology of individuals in regions that vary broadly (like in Chr16p13.11) or specifically (like in the *RPSA* gene or Chr1p13.3 region). To mitigate RAB effectively, it is imperative to align to a genome that is as representative as possible of the true genomes of the individuals under study. This may eventually be done using individual assemblies, as in this study, but that remains cost-prohibitive and requires specialized expertise. A fully complete linear genome, like the telomere-to-telomere human genome assembly T2T-CHM13, is limited by the same issues of representativeness.[8,41] Ultimately, for most researchers, the use of a graph-based genome will be the most viable approach in the long term. Graph-based genomes, such as the draft human pangenome,[16] do not collapse the complexity of multiple individuals into a single string but instead represent variation as paths in a graph. In mapping RAB across genomic contexts, we seek to shed light on the importance of using a representative genome and highlight regions likely to benefit from a graph-based approach.

Guidelines for practitioners

RAB arises from the interaction between local and global differences in genome structure or sequence content and the algorithms used for read alignment. Its effects can vary across samples, assays, and genomic regions, but certain loci are consistently affected across individuals. There are three general strategies to address RAB that differ in feasibility and scope: referencing prior characterizations to inform interpretation, personalizing an existing reference to mitigate bias, and generating individual assemblies to eliminate it entirely.

The first and most immediately applicable strategy is to interpret results in the context of existing RAB characterizations. As more high-quality assemblies and matched functional data become available, it is increasingly possible to identify regions that are recurrently affected by bias across individuals. To facilitate this, we provide genome-wide annotations of loci exhibiting consistent RAB across hg38 and CHM13. These resources can be accessed through the WashU Epigenome Browser as a public data hub (Figure S7A), allowing users to visualize recurrently biased regions and evaluate whether signals of interest overlap genomic areas with a known history of reference-induced bias. An example region on chromosome 1 is shown (Figure S7B), including a detailed view of the *CAP1* gene, where two non-reference transposable element insertions are present, one partial Alu and one complete Alu element (Figure S7C). The presence of these non-reference insertions highlights how structural differences between the reference and individual genomes can distort alignment and coverage patterns. In practice, if a candidate feature falls within a region consistently affected by RAB, additional scrutiny or alternative validation approaches should be considered, whereas candidates in regions with no evidence of bias can be interpreted with greater confidence.

If such cross-referencing indicates that features of interest are likely to be affected by reference bias, a next step is to mitigate it directly through personalization. This second strategy involves incorporating known variants from the individual or population of interest into an existing reference, thereby reducing mismatches that distort alignment and signal. While personalization

can substantially improve accuracy in divergent regions, it remains dependent on accurate genotyping and on the ability to represent variants within the reference framework. Recent advances in reference-quality assemblies and pangenomic resources have made it increasingly feasible to generate or derive personalized references, extending this option to a wider range of samples and assays.

In cases where personalization is insufficient or where structural differences between the sample and reference are extensive, a more direct strategy is to generate a high-quality individual assembly. Aligning reads to an individual's own genome greatly reduces reference-induced artifacts by capturing the individual's true genomic structure, though it cannot eliminate bias entirely given the limitations of current assembly and alignment methods. While this approach remains resource-intensive and impractical for large cohorts, examples such as CHM13 demonstrate how improvements in completeness and structural resolution substantially reduce bias within complex loci, emphasizing the importance of continued refinement of reference assemblies.

Finally, researchers should consider whether their biological system of interest is among those known to exhibit high rates of genetic variation, such as immune, neuronal, and reproductive processes. Results arising from such regions are more likely to overlap sites of recurrent RAB and should therefore be interpreted with additional caution. By contrast, loci with no evidence of bias across individuals and lacking association with hypervariable biological processes can generally be interpreted with greater confidence.

Limitations of the study

We acknowledge multiple technical limitations in our study. First, our method of phasing reads using parental assemblies is limited by MAPQ scores, which measure both mappability and read quality.[20]

Second, the amount of RAB attributed to a specific genomic bin is not independent of other genomic locations, and our current methodological implementation does not account for the effects of copy number variants.

RESOURCE AVAILABILITY

Lead contact

Further information and requests for resources should be directed to and will be fulfilled by the lead contact, Ting Wang (twang@wustl.edu).

Materials availability

This study did not generate new, unique reagents.

Data and code availability

- Raw ATAC-seq and RNA-seq data are available in the Sequence Read Archive Bioproject PRJNA1130678. WGBS data are available via the NCBI Gene Expression Omnibus under accession GSE261315. Alignments are available at https://wangcluster.wustl.edu/public/RAB_Benchmarking/. Washu Epigenome Browser tracks of cumulative RAB across individuals can be accessed for hg38 at https://epigenomegateway.wustl.edu/browser2022/?genome=hg38&position=chr6:24356575-29246084&hub=https://wangcluster.wustl.edu/public/RAB_Benchmarking/hg38_RAB.config.json and for CHM13 at https://epigenomegateway.wustl.edu/browser2022/?genome=t2t-chm13-v1.1&position=chr7:100815966-108432979&hub=https://wangcluster.wustl.edu/public/RAB_Benchmarking/CHM13_RAB.config.json. These resources can be accessed by entering the above links into a web browser. Individual files used to generate the above data hubs can be accessed at https://wangcluster.wustl.edu/public/RAB_Benchmarking/bedGraphs/.
- For the specific commands used in this analysis, see https://doi.org/10.5281/zenodo.19615987 or, on GitHub, https://github.com/twlab/ReferenceAlignmentBias
- Any additional information required to re-analyze the data reported in this paper is available from the lead contact upon request

ACKNOWLEDGMENTS

We would like to thank B. Koebbe and E. Martin from CGSSB for assistance with the processing of data. This work was funded in part by National Institutes of Health (NIH) grants U41HG010972, U41HG010971, U24HG012070, and R01HG007175.

AUTHOR CONTRIBUTIONS

N.T., J.F.M.-V., and T.W. conceived of and implemented the study. J.E.G. and W.Z. assisted with WGBS and RNA-seq analysis. D.A. assisted in genome scaffolding and visualizing results. E.J.B. and C.T. assisted with data and pipeline management. N.T., J.F.M.-V., and T.W. prepared and revised the manuscript.

DECLARATION OF INTERESTS

The authors declare no competing interests.

STAR★METHODS

Detailed methods are provided in the online version of this paper and include the following:

- KEY RESOURCES TABLE
- EXPERIMENTAL MODEL AND STUDY PARTICIPANT DETAILS
  - Samples and genome assembly process
- METHOD DETAILS
  - Generation of genome assemblies
  - Phasing of functional genomic reads
  - Over-representation analysis
  - RNA-seq library preparation and sequencing
  - Whole-genome bisulfite library preparation and sequencing
  - ATAC-seq library preparation
  - Gene annotations preparation
  - RNA-seq index building
  - RNA-seq alignment of phased reads
  - ATAC-seq AIAP genome preparation
  - ATAC-seq phased read alignment
  - WGBS genome index preparation
  - Alignment and quality control of WGBS reads
  - Alignment of parental assemblies to hg38 and CHM13
- QUANTIFICATION AND STATISTICAL ANALYSIS
  - Measuring reference bias
  - Quantifying bias across genomic space

SUPPLEMENTAL INFORMATION

Supplemental information can be found online at https://doi.org/10.1016/j.crmeth.2026.101461.

Received: March 30, 2025
Revised: April 7, 2026
Accepted: April 24, 2026
Published: May 14, 2026

REFERENCES

1. Buenrostro, J.D., Wu, B., Chang, H.Y., and Greenleaf, W.J. (2015). ATAC-seq: A Method for Assaying Chromatin Accessibility Genome-Wide. Curr. Protoc. Mol. Biol. *109*, 21.29.1.1. https://doi.org/10.1002/0471142727.mb2129s109.
2. Wang, Y., Mashock, M., Tong, Z., Mu, X., Chen, H., Zhou, X., Zhang, H., Zhao, G., Liu, B., and Li, X. (2020). Changing Technologies of RNA Sequencing and Their Applications in Clinical Oncology. Front. Oncol. *10*, 447. https://doi.org/10.3389/fonc.2020.00447.
3. Wang, X., and Clark, a G. (2014). Using next-generation RNA sequencing to identify imprinted genes. Heredity *113*, 156–166. https://doi.org/10.1038/hdy.2014.18.
4. Xie, W., Barr, C.L., Kim, A., Yue, F., Lee, A.Y., Eubanks, J., Dempster, E.L., and Ren, B. (2012). Base-Resolution Analyses of Sequence and Parent-of-Origin Dependent DNA Methylation in the Mouse Genome. Cell *148*, 816–831. https://doi.org/10.1016/j.cell.2011.12.035.
5. Lander, E.S., Linton, L.M., Birren, B., Nusbaum, C., Zody, M.C., Baldwin, J., Devon, K., Dewar, K., Doyle, M., Fitzhugh, W., et al. (2001). Initial sequencing and analysis of the human genome. Nature *409*, 860–921. https://doi.org/10.1038/35057062.
6. Frequently Asked Questions - Genome Reference Consortium https://www.ncbi.nlm.nih.gov/grc/help/faq/#dna-source-of-human-reference-genome.
7. Wang, T., Antonacci-Fulton, L., Howe, K., Lawson, H.A., Lucas, J.K., Phillippy, A.M., Popejoy, A.B., Asri, M., Carson, C., Chaisson, M.J.P., et al. (2022). The Human Pangenome Project: a global resource to map genomic diversity. Nature, 437–437446. https://doi.org/10.1038/s41586-022-04601-8.
8. Nurk, S., Koren, S., Rhie, A., Rautiainen, M., Bzikadze, A.V., Mikheenko, A., Vollger, M.R., Altemose, N., Uralsky, L., Gershman, A., et al. (2022). The complete sequence of a human genome. Science *376*, 44–53. https://doi.org/10.1126/science.abj6987.
9. Need, A.C., and Goldstein, D.B. (2009). Next generation disparities in human genomics: concerns and remedies. Trends Genet. *25*, 489–494. https://doi.org/10.1016/j.tig.2009.09.012.
10. Lunter, G., and Goodson, M. (2011). Stampy: A statistical algorithm for sensitive and fast mapping of Illumina sequence reads. Genome Res. *21*, 936–939. https://doi.org/10.1101/gr.111120.110.
11. Lin, M.J., Iyer, S., Chen, N.C., and Langmead, B. (2024). Measuring, visualizing, and diagnosing reference bias with biastools. Genome Biol. *25*, 101. https://doi.org/10.1186/S13059-024-03240-8.
12. Groza, C., Chen, X., Pacis, A., Simon, M.M., Pramatarova, A., Aracena, K.A., Pastinen, T., Barreiro, L.B., and Bourque, G. (2023). Genome graphs detect human polymorphisms in active epigenomic state during influenza infection. Cell Genom. *3*, 100294. https://doi.org/10.1016/j.xgen.2023.100294.
13. Hill, W.G., Goddard, M.E., and Visscher, P.M. (2008). Data and Theory Point to Mainly Additive Genetic Variance for Complex Traits. PLoS Genet. *4*, e1000008. https://doi.org/10.1371/journal.pgen.1000008.
14. Visscher, P.M., Hill, W.G., and Wray, N.R. (2008). Heritability in the genomics era - Concepts and misconceptions. Nat. Rev. Genet. *9*, 255–266. https://doi.org/10.1038/nrg2322;kwrd.
15. Chen, N.C., Solomon, B., Mun, T., Iyer, S., and Langmead, B. (2021). Reference flow: reducing reference bias using multiple population genomes. Genome Biol. *22*, 8–17. https://doi.org/10.1186/S13059-020-02229-3.
16. Liao, W.W., Asri, M., Ebler, J., Doerr, D., Haukness, M., Hickey, G., Lu, S., Lucas, J.K., Monlong, J., Abel, H.J., et al. (2023). A draft human pangenome reference. Nature *617*, 312–324. https://doi.org/10.1038/s41586-023-05896-x.
17. Eizenga, J.M., Novak, A.M., Sibbesen, J.A., Heumos, S., Ghaffaari, A., Hickey, G., Chang, X., Seaman, J.D., Rounthwaite, R., Ebler, J., et al. (2020). Pangenome Graphs. Annu. Rev. Genomics Hum. Genet. *21*, 139–162. https://doi.org/10.1146/annurev-genom-120219-080406.
18. Clarke, L., Zheng-Bradley, X., Smith, R., Kulesha, E., Xiao, C., Toneva, I., Vaughan, B., Preuss, D., Leinonen, R., Shumway, M., et al. (2012). The 1000 Genomes Project: data management and community access. Nat. Methods *9*, 459–462. https://doi.org/10.1038/nmeth.1974.
19. Auton, A., Abecasis, G.R., Altshuler, D.M., Durbin, R.M., Bentley, D.R., Chakravarti, A., Clark, A.G., Donnelly, P., Eichler, E.E., Flicek, P., et al. (2015). A global reference for human genetic variation. Nature *526*, 68–74. https://doi.org/10.1038/nature15393.
20. Li, H., Handsaker, B., Wysoker, A., Fennell, T., Ruan, J., Homer, N., Marth, G., Abecasis, G., and Durbin, R.; 1000 Genome Project Data Processing Subgroup (2009). The Sequence Alignment/Map format and SAMtools. Bioinformatics *25*, 2078–2079. https://doi.org/10.1093/bioinformatics/btp352.
21. Martin, M., Patterson, M., Garg, S., O Fischer, S., Pisanti, N., Klau, G.W., Schöenhuth, A., and Marschall, T. (2016). WhatsHap: fast and accurate read-based phasing. Preprint at bioRxiv. https://doi.org/10.1101/085050.
22. Daniel, W.W. (1990). Applied Nonparametric Statistics. 635
23. R: The R Project for Statistical Computing https://www.r-project.org/.
24. Wickham, H. (2009). ggplot2. ggplot2. https://doi.org/10.1007/978-0-387-98141-3.
25. Gu, Z., Gu, L., Eils, R., Schlesner, M., and Brors, B. (2014). Circlize implements and enhances circular visualization in R. Bioinformatics *30*, 2811–2812. https://doi.org/10.1093/BIOINFORMATICS/BTU393.
26. Li, D., Purushotham, D., Harrison, J.K., Hsu, S., Zhuo, X., Fan, C., Liu, S., Xu, V., Chen, S., Xu, J., et al. (2022). WashU Epigenome Browser update 2022. Nucleic Acids Res. *50*, W774–W781. https://doi.org/10.1093/NAR/GKAC238.
27. Zhuo, X., Hsu, S., Purushotham, D., Kuntala, P.K., Harrison, J.K., Du, A.Y., Chen, S., Li, D., and Wang, T. (2023). Comparing genomic and epigenomic features across species using the WashU Comparative Epigenome Browser. Genome Res. *33*, 824–835. https://doi.org/10.1101/GR.277550.122.
28. Porubsky, D., Guitart, X., Yoo, D.A., Dishuck, P.C., Harvey, W.T., and Eichler, E.E. (2025). SVbyEye: a visual tool to characterize structural variation among whole-genome assemblies. Bioinformatics *41*. https://doi.org/10.1093/BIOINFORMATICS/BTAF332.
29. Frankish, A., Diekhans, M., Ferreira, A.M., Johnson, R., Jungreis, I., Loveland, J., Mudge, J.M., Sisu, C., Wright, J., Armstrong, J., et al. (2019). GENCODE reference annotation for the human and mouse genomes. Nucleic Acids Res. *47*, D766–D773. https://doi.org/10.1093/NAR/GKY955.
30. Feingold, E.A., Good, P.J., Guyer, M.S., Kamholz, S., Liefer, L., Wetterstrand, K., Collins, F.S., Gingeras, T.R., Kampa, D., Sekinger, E.A., et al. (2004). The ENCODE (ENCyclopedia of DNA Elements) Project. Science *306*, 636–640. https://doi.org/10.1126/SCIENCE.1105136.
31. Semple, F., and Dorin, J.R. (2012). β-Defensins: Multifunctional Modulators of Infection, Inflammation and More? J. Innate Immun. *4*, 337–348. https://doi.org/10.1159/000336619.
32. Quintana-Murci, L., and Clark, A.G. (2013). Population genetic tools for dissecting innate immunity in humans. Nat. Rev. Immunol. *13*, 280–293. https://doi.org/10.1038/NRI3421.
33. Trimmer, C., Keller, A., Murphy, N.R., Snyder, L.L., Willer, J.R., Nagai, M.H., Katsanis, N., Vosshall, L.B., Matsunami, H., and Mainland, J.D. (2019). Genetic variation across the human olfactory receptor repertoire alters odor perception. Proc. Natl. Acad. Sci. USA *116*, 9475–9480. https://doi.org/10.1073/PNAS.1804106115.
34. Tran, Q., Gao, S., and Phan, V. (2016). Analysis of optimal alignments unfolds aligners' bias in existing variant profiles. BMC Bioinf. *17*, 349. https://doi.org/10.1186/S12859-016-1216-1.
35. Holdt, L.M., and Teupser, D. (2013). From genotype to phenotype in human atherosclerosis - Recent findings. Curr. Opin. Lipidol. *24*, 410–418. https://doi.org/10.1097/MOL.0B013E3283654E7C.

36. Martin, J., Han, C., Gordon, L.A., Terry, A., Prabhakar, S., She, X., Xie, G., Hellsten, U., Chan, Y.M., Altherr, M., et al. (2004). The Sequence and Analysis of Duplication-Rich Human Chromosome 16. Nature *432*, 988. https://doi.org/10.1038/nature03187.

37. Ramalingam, A., Zhou, X.G., Fiedler, S.D., Brawner, S.J., Joyce, J.M., Liu, H.Y., and Yu, S. (2011). 16p13.11 duplication is a risk factor for a wide spectrum of neuropsychiatric disorders. J. Hum. Genet. *56*, 541–544. https://doi.org/10.1038/jhg.2011.42.

38. Cantsilieris, S., Sunkin, S.M., Johnson, M.E., Anaclerio, F., Huddleston, J., Baker, C., Dougherty, M.L., Underwood, J.G., Sulovari, A., Hsieh, P., et al. (2020). An evolutionary driver of interspersed segmental duplications in primates. Genome Biol. *21*, 202. https://doi.org/10.1186/S13059-020-02074-4.

39. Bailey, J.A., and Eichler, E.E. (2006). Primate segmental duplications: Crucibles of evolution, diversity and disease. Nat. Rev. Genet. *7*, 552. https://doi.org/10.1038/nrg1895.

40. Gittelman, R.M., Hun, E., Ay, F., Madeoy, J., Pennacchio, L., Noble, W.S., Hawkins, R.D., and Akey, J.M. (2015). Comprehensive identification and analysis of human accelerated regulatory DNA. Genome Res. *25*, 1245–1255. https://doi.org/10.1101/GR.192591.115.

41. Aganezov, S., Yan, S.M., Soto, D.C., Kirsche, M., Zarate, S., Avdeyev, P., Taylor, D.J., Shafin, K., Shumate, A., Xiao, C., et al. (2022). A complete reference genome improves analysis of human genetic variation. Science *376*, eabl3533. https://doi.org/10.1126/science.abl3533.

42. Alonge, M., Lebeigle, L., Kirsche, M., Jenike, K., Ou, S., Aganezov, S., Wang, X., Lippman, Z.B., Schatz, M.C., and Soyk, S. (2022). Automated assembly scaffolding using RagTag elevates a new tomato system for high-throughput genome editing. Genome Biol. *23*, 258. https://doi.org/10.1186/S13059-022-02823-7.

43. Picard Tools - By Broad Institute https://broadinstitute.github.io/picard/.

44. Cline, E., Wisittipanit, N., Boongoen, T., Chukeatirote, E., Struss, D., and Eungwanichayapant, A. (2020). Recalibration of mapping quality scores in Illumina short-read alignments improves SNP detection results in low-coverage sequencing data. PeerJ *8*, e10501. https://doi.org/10.7717/PEERJ.10501/SUPP-8.

45. Dobin, A., Davis, C.A., Schlesinger, F., Drenkow, J., Zaleski, C., Jha, S., Batut, P., Chaisson, M., and Gingeras, T.R. (2013). STAR: ultrafast universal RNA-seq aligner. Bioinformatics *29*, 15–21. https://doi.org/10.1093/BIOINFORMATICS/BTS635.

46. Krueger, F., and Andrews, S.R. (2011). Bismark: a flexible aligner and methylation caller for Bisulfite-Seq applications. Bioinformatics *27*, 1571–1572. https://doi.org/10.1093/BIOINFORMATICS/BTR167.

47. Heinz, S., Benner, C., Spann, N., Bertolino, E., Lin, Y.C., Laslo, P., Cheng, J.X., Murre, C., Singh, H., and Glass, C.K. (2010). Simple Combinations of Lineage-Determining Transcription Factors Prime cis-Regulatory Elements Required for Macrophage and B Cell Identities. Mol. Cell *38*, 576–589. https://doi.org/10.1016/j.molcel.2010.05.004.

48. Corces, M.R., Trevino, A.E., Hamilton, E.G., Greenside, P.G., Sinnott-Armstrong, N.A., Vesuna, S., Satpathy, A.T., Rubin, A.J., Montine, K.S., Wu, B., et al. (2017). An improved ATAC-seq protocol reduces background and enables interrogation of frozen tissues. Nat. Methods *14*, 959–962. https://doi.org/10.1038/nmeth.4396.

49. Shumate, A., and Salzberg, S.L. (2021). Liftoff: accurate mapping of gene annotations. Bioinformatics *37*, 1639–1643. https://doi.org/10.1093/BIOINFORMATICS/BTAA1016.

50. Hitz, B.C., Lee, J.-W., Jolanki, O., Kagda, M.S., Graham, K., Sud, P., Gabdank, I., Strattan, J.S., Sloan, C.A., Dreszer, T., et al. (2023). The ENCODE Uniform Analysis Pipelines. Res. Sq. https://doi.org/10.21203/RS.3.RS-3111932/V1.

51. mrvollger/rustybam: bioinformatics toolkit in rust https://github.com/mrvollger/rustybam.

52. Martin, M. (2011). Cutadapt removes adapter sequences from high-throughput sequencing reads. EMBnet J. *17*, 10–12. https://doi.org/10.14806/EJ.17.1.200.

53. Langmead, B., and Salzberg, S.L. (2012). Fast gapped-read alignment with Bowtie 2. Nat. Methods *9*, 357–359. https://doi.org/10.1038/nmeth.1923.

54. Li, H. (2018). Minimap2: pairwise alignment for nucleotide sequences. Bioinformatics *34*, 3094–3100. https://doi.org/10.1093/BIOINFORMATICS/BTY191.

55. Li, H. (2013). Aligning Sequence Reads, Clone Sequences and Assembly Contigs with BWA-MEM. Preprint at arXiv. https://doi.org/10.48550/arXiv.1303.3997.

56. Zhang, B., Kirov, S., and Snoddy, J. (2005). WebGestalt: An integrated system for exploring gene sets in various biological contexts. Nucleic Acids Res. *33*, W741–W748. https://doi.org/10.1093/nar/gki475.

57. Li, B., and Dewey, C.N. (2011). RSEM: Accurate transcript quantification from RNA-Seq data with or without a reference genome. BMC Bioinf. *12*, 323. https://doi.org/10.1186/1471-2105-12-323.

58. Babraham Bioinformatics - FastQC A Quality Control tool for High Throughput Sequence Data https://www.bioinformatics.babraham.ac.uk/projects/fastqc/.

59. Liu, S., Li, D., Lyu, C., Gontarz, P.M., Miao, B., Madden, P.A.F., Wang, T., and Zhang, B. (2021). AIAP: A Quality Control and Integrative Analysis Package to Improve ATAC-Seq Data Analysis. Genom. Proteom. Bioinform. *19*, 641–651. https://doi.org/10.1016/J.GPB.2020.06.025.

60. Quinlan, A.R., and Hall, I.M. (2010). BEDTools: a flexible suite of utilities for comparing genomic features. Bioinformatics *26*, 841–842. https://doi.org/10.1093/BIOINFORMATICS/BTQ033.

61. Sthle, L., and Wold, S. (1989). Analysis of variance (ANOVA). Chemometr. Intell. Lab. Syst. *6*, 259–272. https://doi.org/10.1016/0169-7439(89)80095-4.

62. Ferreira, J.A., and Zwinderman, A.H. (2006). On the Benjamini–Hochberg Method. Ann. Statist. *34*, 1827–1849. https://doi.org/10.1214/009053606000000425.

CellPress
OPEN ACCESS

STAR★METHODS

KEY RESOURCES TABLE

REAGENT or RESOURCE	SOURCE	IDENTIFIER
Deposited data		
Raw RNA-seq and ATAC-seq	NCBI	SRA: PRJNA1130678
Raw WGBS	NCBI	GEO: GSE261315
GRCh38	Genome Reference Consortium	https://www.ncbi.nlm.nih.gov/datasets/genome/GCF_000001405.40/
CHM13 v1.1	Telemore-to-Telomere Consortium	https://www.ncbi.nlm.nih.gov/datasets/genome/GCA_009914755.3/
RAB genome browser tracks	This paper	https://wangcluster.wustl.edu/public/RAB_Benchmarking/bedGraphs/
Software and algorithms		
Samtools 1.14	Li et al.[20]	https://www.htslib.org/
WashU Epigenome Browser	Li et al.[25]	https://epigenomegateway.wustl.edu/
RagTag 2.1.0	Alonge et al.[28]	https://github.com/malonge/RagTag
Picard 0.2.4	Broad Institute[42]	https://github.com/broadinstitute/picard
BWA-mem 0.7.17	Li[43]	https://github.com/lh3/bwa
STAR 2.9.7a	Dobin et al.[44]	https://github.com/alexdobin/STAR
Bismark 0.23.1	Krueger and Andrews[45]	https://www.bioinformatics.babraham.ac.uk/projects/bismark/
HOMER 5.1	Heinz et al.[46]	http://homer.ucsd.edu/homer/
WebGestalt	Zhang et al.[47]	https://www.webgestalt.org/
Liftoff 1.6.3.6	Shumate and Salzberg[48]	https://github.com/agshumate/Liftoff
RSEM 1.2.31	Li and Dewey[49]	https://github.com/deweylab/RSEM
Rustybam 0.1.29	Vollger[50]	https://github.com/mrvollger/rustybam
Cutadapt 2.10	Martin[51]	https://github.com/marcelm/cutadapt/
FastQC 0.11.9	Babraham Bioinformatics[52]	https://www.bioinformatics.babraham.ac.uk/projects/fastqc/
Minimap2 2.24	Li[53]	https://github.com/lh3/minimap2
PAFtools 2.24	Li[53]	https://github.com/lh3/minimap2
Bedtools 2.30.0	Quinlan and Hall[54]	https://github.com/arq5x/bedtools2
Scripts and commands used in this study	This study	https://github.com/twlab/ReferenceAlignmentBias

EXPERIMENTAL MODEL AND STUDY PARTICIPANT DETAILS

Samples and genome assembly process

In this study, we employed immortalized lymphoblastoid cell lines (LCLs) from the 1000 Genomes Project,[18] which were subsequently utilized by the Human Pangenome Reference Consortium (HPRC) for generating comprehensive genomic data. Our focus was on a subset of these LCLs, specifically HG01952 (Male, Peruvian), HG00741 (Female, Puerto Rican), HG00621 (Male, Han Chinese South), HG01978 (Female, Peruvian), and HG03516 (Female, Esan).

METHOD DETAILS

Generation of genome assemblies

Assemblies were generated by the Human Pangenome Reference Consortium (HPRC) as part of the Draft Human Pangenome Project, following the methodology previously described.[16] Briefly, high-quality phased diploid assemblies were produced using PacBio HiFi long-read sequencing, Omni-C Hi-C data, and parental short-read sequencing with Hifiasm yielding haplotype-resolved contig-level assemblies. These assemblies are publicly available through NCBI FTP sites. For downstream analyses, contig-level FASTA files were indexed with Samtools (v1.14) and scaffolded against the telomere-to-telomere reference genome (T2T-CHM13).

T2T-CHM13v1.1 was used for most assemblies, while T2T-CHM13v2.0 (GCA_009914755.4) was used for the paternal assemblies HG00621 and HG01952 to include the Y chromosome. Scaffolding was performed with RagTag (v2.1.0),[42] followed by post-processing to remove suffixes, split primary and unplaced sequences, and standardize chromosome headers. Primary sequences were reordered in canonical order (chr1–chr22, chrX, chrY, chrM) and normalized with Picard (v0.2.4).[43] Scaffold statistics, including placed and unplaced sequences, gap counts, and gap base pairs, were extracted from RagTag output. Gap and sequence length analyses were performed on both contig-level and RagTag-scaffolded assemblies using a custom python script, which reports gap coordinates (BED format) and per-chromosome statistics. Scaffold N50 values were obtained from NCBI Genome Navigator. Assembly QC values can be seen in Table S4.

Phasing of functional genomic reads

To quantify reference bias, it is assumed that aligning reads derived from an individual to the individual's own genome will indicate their true placement. However, functional genomics assays produce data consisting of a mixture of sequenced fragments (reads) originating from either the maternal or paternal genomes. Deconvoluting the parental origins of reads is essential when measuring reference bias because a failure to do so leads to artifacts produced by forcing the alignment of reads derived from one parent to the genome from the second parent. Phasing in this context is the sorting of reads by parental origins.

Phasing was applied across all three assays. Reads were aligned separately to the maternal, paternal, and combined genome assemblies for a comprehensive comparison. By comparing preferential alignment to one parent or the other, we can assign reads to either the maternal or paternal genome. Any read differentially attributed to a parental genome resulted in its paired read also being attributed to the same parental genome.

Alignment of ATAC-seq data to all three genome assemblies was performed using the BWA-MEM aligner.[55] Phasing of reads was determined by evaluating the Mapping Quality (MAPQ) score for each alignment. MAPQ is defined as $-10\log10(p)$, where p is the probability the read was misaligned.[44] For any given read, if an alignment in the combined genome had a resulting MAPQ of greater than 0, then it was mapped with higher confidence to one location than the other and was placed differentially in the parental genome it was aligned to. Reads with a MAPQ score of 0, indicative of multi-mapping, were attributed to both parental genomes unless unmapped in either parental assembly. All other reads, regardless of whether they were mapped or not, were attributed to both assemblies.

RNA-seq data was aligned using the STAR aligner.[45] Reads were phased using both MAPQ scores and secondary alignments. For any given read, if it had no secondary alignment, then it was attributed differentially to the parent of the primary alignment. If it had a secondary alignment location in the combined alignment, it was attributed to both parental assemblies if the MAPQ scores were equivalent; otherwise, it was attributed differentially to the parent of the primary alignment. All other reads were attributed to both parental assemblies.

WGBS data alignment was conducted with the Bismark aligner[46] (v0.23.1). Bismark alignments do not include multi-mapped reads; thus, any read that was mapped in the combined alignment was attributed to the parental assembly to which it aligned. All other reads were attributed to both.

Over-representation analysis

Bins were stratified for over-representation analysis (ORA) by technology and classification, resulting in 6 strata, and by technology, classification, individual, and haplotype, resulting in 60 ($3 \times 2 \times 5 \times 2$) strata, for a total of 66 analyzed strata. For each stratum, all bins with alignments/signal >10 were used as inputs to HOMER (v5.1),[47] for annotation with genomic features. Custom scripts were used to extract ensembl gene IDs from bins annotated as intersecting promoters. Genes in bins exhibiting RAB were submitted to WebGestalt[56] for ORA, with genes from all bins used as background. 5 databases were used for analysis: cytogeneic bands, Gene Ontology (GO) biological process, GO cellular component, GO molecular function, and Kyoto Encyclopedia of Genes and Genomes (KEGG) pathway. False discovery rate (FDR) was calculated using the Benjamini–Hochberg procedure.

RNA-seq library preparation and sequencing

Total RNA integrity was evaluated using an Agilent Bioanalyzer. Library preparation was performed using 500 ng to 1 μg of total RNA. Ribosomal RNA removal was conducted via an RNase-H-based method, specifically using RiboErase kits from Kapa Biosystems. Following ribosomal RNA depletion, mRNA was fragmented by incubation in reverse transcriptase buffer and subsequent heating at 94°C for eight minutes. This fragmented mRNA was reverse transcribed into cDNA using the SuperScript III Reverse Transcriptase enzyme from Life Technologies, in accordance with the manufacturer's protocols, and employing random hexamers. A second-strand cDNA synthesis was performed, converting the single-stranded cDNA into double-stranded cDNA (ds-cDNA). The ds-cDNA was then subjected to end repair processes to create blunt ends, followed by the addition of an adenine base to the 3′ ends. Illumina sequencing adapters, which contained unique dual index tags, were ligated to these prepared ends. The adapter-ligated cDNA fragments underwent PCR amplification for 12 to 15 cycles using primers that included unique dual index tags. Sequencing was performed on an Illumina NovaSeq 6000 system, producing paired-end reads that extended 150 bases in length. It is important to note that the resultant sequencing data is unstranded.

Whole-genome bisulfite library preparation and sequencing

The process of library preparation and sequencing was conducted at the McDonnell Genome Institute's Genome Technology Access Center (GTAC). Initially, genomic DNA (gDNA) was quantified utilizing the Qubit 1× HS dsDNA assay kit (Invitrogen, Cat# Q33231). The library preparation used 200 ng of gDNA, to which 0.2% unmethylated Lambda DNA was added as a control to assess the efficiency of bisulfite conversion. This mixture was then fragmented into approximately 350 bp segments using the Covaris LE220, followed by a 1.5× AMPure purification step on the EpMotion 5075 (Eppendorf), yielding a final volume of 20 μL. The gDNA was then subjected to bisulfite conversion employing the EZ-96 DNA Methylation-Gold Mag Prep Kit (Zymo Research, Cat# D5043) in accordance with the protocols recommended by the manufacturer, executed on the EpMotion 5075. The converted DNA (bsDNA) was quantified employing the Qubit ssDNA Assay Kit (Thermo Fisher Scientific, Cat# Q10212). The construction of automated WGBS libraries utilized roughly 100 ng of this quantified bsDNA, leveraging the Accel-NGS Methyl-Seq DNA Library Kit (Swift BioSciences, Cat# 30096) combined with unique dual indexes (UDI) (Swift, ×9096) on the EpMotion 5075. The indexing PCR step comprised eight cycles, followed by a 0.85× AMPure cleanup to finalize the library. Assessment of the final library metrics, including average size and concentration, was conducted using a GX instrument. Precise quantification before sequencing was achieved using qPCR with the KAPA Library Quantification Kits (Roche) to ensure appropriate cluster densities. Sequencing was then performed to yield 2 × 150 paired-end reads using the S4 300 Cycle kit with the XP workflow on Illumina NovaSeq 6000 systems.

ATAC-seq library preparation

Nuclei were isolated from frozen tissues in accordance with a modified protocol developed by the Howard Chang Lab.[48] Initially, frozen cell samples, weighing between 50 and 100 mg (~50,000 cells), were mechanically disrupted into a fine powder using a Cellcrusher in liquid nitrogen to prevent thawing. Around 20 mg of this powdered tissue was then precisely measured and transferred into a chilled 1.5 mL tube with a pre-cooled spatula to ensure a consistent sample volume. The tissue was then resuspended in 1 mL of ice-cold Nuclei Isolation Buffer (NIB) devoid of detergent to mitigate mitochondrial contamination. This mixture was gently mixed by inversion and placed on wet ice for homogenization on an orbital shaker set at 120 rpm for five minutes. Following homogenization, the suspension was filtered through a 100 μm CellTrics filter and centrifuged at 1100 × g for ten minutes at 4°C to pellet the nuclei, which were then resuspended in 50 μL of RSB buffer through gentle pipetting. Density gradient centrifugation was employed for further purification using a layered iodixanol solution, ensuring minimal disruption to the density layers. Post-centrifugation, the nuclei were located at the interface between the 29% and 35% iodixanol layers, carefully extracted, and washed in cold ATAC-RSB supplemented with 0.1% Tween 20. For ATAC-seq library preparation, approximately 50,000 nuclei were resuspended in 25 μL of 2× TD buffer and combined with the Omni-ATAC ATAC-seq reaction mix. This mixture underwent incubation at 37°C for 30 min with shaking at 1000 RPM. Post-transposition, the DNA was purified using the Zymo DNA Clean and Concentrator-5 kit to avoid cross-contamination. The purified, transposed DNA was amplified using NEBNext 2× MasterMix and Nextera primers under specific cycling conditions. Following PCR amplification, the library underwent a size-selection process using Ampure XP beads to isolate DNA fragments predominantly ranging from 100 to 500 bp. The final library concentration was quantified using the Qubit dsDNA HS Assay Kit, and the size distribution was assessed using a 4200 TapeStation with High Sensitivity D1000 ScreenTape and Reagents. Sequencing was then performed to yield 2 × 75 paired-end reads using the Illumina NextSeq platform.

Gene annotations preparation

The Gene Transfer Format (GTF) files required for the analysis were generated through a process utilizing Liftoff (version 1.6.3.6).[49] Liftoff is a tool designed for the annotation of novel genome assemblies by transferring known annotations from a reference genome to the target genome. In our study, the target genome assemblies (diploid maternal and paternal) were provided in FASTA format. The procedure commenced with the preparation of the necessary input files: the target genome assembly FASTA files and the reference annotations (hg38 FASTA and Gencode v29 GTF). The tool Liftoff was run to align the reference annotations to the corresponding sequences in the target genome assemblies. This alignment process is critical, as it enables the precise transfer of gene structures, including exons, introns, and other genomic elements, from the reference to the target genomes while accommodating the sequence differences between them. This produced GTF files for each target genome assembly.

RNA-seq index building

Prior to alignment, necessary indices were constructed for the alignment and quantification tools: STAR (version 2.7.9a)[45] and RSEM (version 1.2.31),[57] following the ENCODE pipeline modifications for custom genome assemblies.[50] For hg38, prebuilt index files were acquired from the ENCODE repository. For individual genomes, indices were generated by merging annotation GTF files with ERCC and phiX spike-in sequences, excluding tRNA annotations for this analysis. STAR indices were prepared using the genomeGenerate mode, supplying merged GTF, target genome FASTA, and spike-ins, and setting the sjdbOverhang parameter to one less than the read length. RSEM indices were similarly prepared, aligning to the target genome and merged annotation GTF.

RNA-seq alignment of phased reads

The phased maternal and paternal RNA-seq sets were each aligned against their corresponding parental assembly (i.e., maternal reads aligned to the maternal assembly) in addition to the hg38/CHM13 assembly. Alignment was performed using the STAR aligner, adhering to the parameters outlined in the ENCODE protocol and adjusted for specific read lengths and insert sizes. The alignments were

processed against the custom-built STAR index's included options for quantMode to facilitate downstream RSEM analysis. Outputs included sorted BAM files and associated metrics, transcriptome-aligned BAM for RSEM input, unmapped reads in FASTQ format, and comprehensive log files. Post-alignment QC was executed using Picard CollectRnaSeqMetrics, analyzing the distribution of reads across genomic regions (e.g., exons, introns, UTRs, and intergenic spaces) to assess library quality.[43] Additional QC metrics were compiled, including read counts by gene type categories and comprehensive alignment statistics provided by SAMtools flagstat.[20] The pipeline also integrated a BAM to signals step, converting BAM files to bigWig format for visual inspection of alignment distribution.

ATAC-seq AIAP genome preparation

Genomic indexes and annotation files (used for QC) are essential for accurate alignment and interpretation of sequencing data. BWA (Burrows-Wheeler Aligner) indexes were generated utilizing all the reference and individual genomes in FASTA format. The mitochondrial genome of each individual was included in both maternal and paternal genomes to allow for quantification of mitochondrial contamination.

The annotation files include chromosome size files delineating the length of each chromosome in the reference genome, as well as promoter and coding promoter BED files. These files are used to evaluate the quality of the ATAC-seq data. The annotations were generated by lifting over the hg38 annotations provided with AIAP to each individual assembly using rustybam and whole genome alignments in PAF format.[51]

ATAC-seq phased read alignment

The phased raw sequencing reads were processed using the cutadapt[52] tool, which trimmed sequencing adapters and low-quality bases. The quality of the processed reads was further evaluated using the FastQC tool,[58] which provided insights into potential quality issues such as overrepresented sequences or abnormal GC content. This step was crucial for ensuring the reliability of the sequencing data.

Cleaned reads were then aligned to the respective genomes (maternal reads to the maternal genome, maternal reads to the hg38|CHM13 genome, paternal reads to the paternal genome, and paternal reads to the hg38|CHM13 genome) using the BWA MEM algorithm to produce alignments in BAM format.

After alignment, reads were subjected to de-duplication to remove duplicate fragments resulting from PCR amplification. Lastly, the pipeline included the calculation of comprehensive quality control metrics.[59] These metrics assessed the overall quality of the sequencing and alignment processes, including sequencing depth, alignment efficiency, and the representation of chromatin accessibility. Run QC metrics were compared to AIAP-TARGETII standards to ensure sufficient library quality. An additional test was performed to measure mitochondrial DNA contamination, a common concern in ATAC-seq datasets due to the abundance of mitochondrial DNA.

WGBS genome index preparation

The alignment of WGBS reads requires bisulfite-specific indexes. Indexes are prepared by converting cytosines to thymines (and guanines to adenines for the reverse strand) in the reference, simulating the bisulfite treatment effect. This index generation is critical for aligning bisulfite-converted reads against the reference. Indexes were constructed using Bismark, a tool developed for bisulfite sequencing data analysis.[46] Indexes were prepared for hg38, CHM13, and each of the individual genome assemblies.

Alignment and quality control of WGBS reads

Raw sequencing reads from WGBS were first subjected to adapter sequence removal and quality trimming using the cutadapt tool. This step is crucial to eliminating sequencing adapters and low-quality bases from the reads, which could otherwise interfere with alignment accuracy and methylation calling. Following trimming, the FastQC tool was employed to assess the quality of the reads. FastQC provided visual and quantitative reports indicating the quality of the sequencing output, allowing for the identification of potential issues that might affect the analysis, such as low-quality ends, over-represented adapters, or contamination. This step ensured that only high-quality reads were forwarded to the alignment stage. The reads were then aligned using Bismark (maternal reads to the maternal genome, maternal reads to the hg38/CHM13 genome, paternal reads to the paternal genome, and paternal reads to the hg38/CHM13 genome). This tool performs a two-step process where bisulfite-treated reads are first converted in silico and then aligned to the bisulfite-converted reference genome indexes. Bismark utilizes Bowtie2 as the underlying alignment engine.[53] The alignment process is optimized to discriminate between methylated and unmethylated cytosines effectively, resulting in the generation of BAM files that detail the alignment positions and methylation states of reads across the genome.

Alignment of parental assemblies to hg38 and CHM13

Each of the individual assemblies was aligned against hg38 and CHM13. Whole genome alignment was performed using minimap2 with the following command[54]:

minimap2 − x asm5 − a − L − − cs = long − t 16 ../ref/hg38.fa $f − o ${f%_ragtag.fasta}.hg38.sam; paftools.js sam2paf − p − L ${f%_ragtag.fasta}.hg38.sam > ${f%_ragtag.fasta}.hg38.paf

The resulting PAF files were used to perform liftover of bins and annotations as well as to visualize alignments across assemblies using the WashU epigenome browser.[27]

QUANTIFICATION AND STATISTICAL ANALYSIS

Measuring reference bias

RAB was quantified across genomic space using a method similar to that employed by Lunter and Goodson, which was specifically developed for assessing RAB in WGS data alignments with the Stampy aligner.[10] This method quantifies a narrow sense of RAB, particularly focusing on variant calling in whole genome sequencing data alignments. They assess the alternative allele fraction (Af) at high-confidence heterozygous and indel sites from a single alignment to hg38.

$$\textit{Alternative Fraction }(Af) = \frac{\textit{query count}}{hg38|CHM13\ \textit{count}+\textit{query count}} \quad \text{(Equation 1)}$$

Assuming equal representation of alleles in WGS data, the expected Af value across the genome should be 0.5, reflecting a 1:1 allelic ratio. The cumulative distribution of Af values is compared to the null expectation in which the CDF inflection point is centered on Af of 0.5. A leftward shift below 0.5 indicates a global preferential alignment to the reference allele, a bias toward the reference. However, the null assumption of a 1:1 allele ratio is not applicable to functional genomics. In functional genomics, individuals may have varying transcript copy numbers, and alignment rates of reads more generally are used to infer continuous functional metrics. Therefore, while the Af metric serves as a bias indicator in WGS, for functional genomics, we adapt the methodology to address the different nature of RAB within these assays.

We define broad-sense reference alignment bias (RAB) as variations in both the quantity and specific identity of reads aligned to corresponding syntenic regions across different reference genomes. To quantify this form of RAB within a particular genomic segment, we introduce a metric termed ‘reference fraction’ (Rf). This metric is derived by independently aligning sequencing reads to two genomic references: the standard reference genome (hg38 or CHM13) and the individual’s assembled (query) genome. For each genomic region under consideration, the ‘reference fraction’ is calculated as the ratio of the number of reads aligned to the reference to the total number of reads aligned to either the reference or the individual’s genome.

$$\textit{Reference Fraction }(Rf) = \frac{hg38|CHM13\ \textit{count}}{hg38|CHM13\ \textit{count}+\textit{query count}} \quad \text{(Equation 2)}$$

For each individual genome assembly, the ‘bedtools’ utility was used to divide the genome into non-overlapping bins of 500 base pairs each.[60] Subsequently, these bins were converted to hg38/CHM13 coordinate space using ‘PAFtools liftover’, using genome-wide alignments between the individual assemblies and hg38/CHM13. This liftover produced bin pairs representing syntenic regions between the individual’s genome (query) and the reference genome (hg38 or CHM13). For each query bin, we keep track of whether it could be lifted over to hg38, if it had reads mapping to it, and, if it did, we compare the length of the syntenic bin pairs (reference versus query). The outcome, or ‘fate’, of bin pairs is indicative of the type of variation present in their originating regions. The different fates of bins are shown in Figure 1B. A query genome bin that failed to liftover suggests it comes from an insertion or unique sequence not present in hg38. Bin pairs devoid of reads are not informative for our analysis. Bin pairs with differing lengths indicate they originate from regions of structural variation. Finally, a pair with equal lengths indicates they originate from regions without structural variation. The likelihood of a 500-bp region having exactly the right combination of structural variants to cancel each other out is low.

The overlap count for each bin was determined using ‘bedtools intersect’, which tallied aligned reads with at least 51% overlap to either bin based on separate alignments from each phased NGS assay to the individual’s genome and to hg38|CHM13. For each pair, the reference fraction was computed using read counts obtained independently from the reference (hg38|CHM13) and query (individual’s genome) bins. Through this process, we identified bin pairs corresponding to syntenic regions across reference and query genomes, associating each with a calculated reference fraction (Figure 1A). It’s important to note that the ‘reference fraction’ serves as a means to measure bias within individual regions. The subsequent analysis compares these measurements against a null expectation for collections of syntenic regions to identify deviations indicative of broad-sense RAB.

Quantifying bias across genomic space

In our approach, the cumulative distribution of the Rf of bin pairs across different levels of genomic space was used as a measure of broad-sense RAB. In a sample that experienced no RAB, the cumulative distribution of reads would fall exclusively within a reference fraction of 0.5. To highlight areas with substantial RAB, we expanded the unbiased reference fraction range from 0.4 to 0.6. Any bin pair that fell outside of this threshold was considered to have reference alignment bias.

This method of quantifying bias assumes that the composition of reads mapped to a given bin pair does not differ between bins when the Rf is 0.5. This assumption permits the quantification of reference bias for a local and genome-wide level as a single value.

Statistical comparisons of Rf distributions versus the null expectation were conducted using the Kolmogorov-Smirnov (KS) statistic.[22,23] This was done at the level of samples, chromosomes, and features. The KS statistic quantifies the difference between the observed CDF and the expected CDF for no bias, in which all bins have a reference fraction of 0.5. Analysis of variance (ANOVA)

and variance partitioning were performed to quantify how RAB varies as a function of individuals, replicates, chromosomes, and genomic contexts.[61] Multiple test corrections were performed using the Benjamini-Hochberg procedure to control for false discovery rate (FDR).[62]

RAB was explored within various genomic annotation sets by mapping their overlapping bins' reference fractions using 'bedtools map', taking the mean Rf of all bins that overlapped with the genomic feature by at least 51% of the annotation or bin. The mean Rf of genomic annotations was calculated separately for bins of unequal lengths and bins of equal lengths (Figure 1C). This methodology enhances our understanding of RAB by aligning measurements with the null expectation and delineating regions where bias diverges significantly from this baseline. It enables a systemic comparison against a theoretical model devoid of bias, identifying genomic spaces particularly affected by RAB and thereby informing the importance of transitioning to a graph-based reference genome.

Cell Reports Methods, Volume *6*

Supplemental information

Quantifying reference alignment bias in functional genomics analyses

Nina Tekkey, John E. Garza, Wenjin Zhang, Derek Albracht, Chad Tomlinson, Edward A. Belter, Xiaoyun Xing, Juan F. Macias-Velasco, and Ting Wang

Supplement

Supplemental Table 1: Bin fate fractions as an average across all five individuals and replicates and fractions of the genome affected by RAB on a chromosome level, as an average across all samples, related to Figures 3 and 4. Left to right, columns are chromosome, parent, technology, reference, mean fraction of unequal bins, standard deviation of fraction of unequal bins, mean fraction of equal bins, standard deviation of fraction of equal bins, mean fraction of bins that did not liftover, standard deviation of fraction of bins that did not liftover, mean fraction of bins that did not have reads, standard deviation of fraction of bins that did not have reads, mean total number of bins, standard deviation of total number of bins, mean of total bins across parents, standard deviation of total bins, mean fraction of bins located on chromosome, mean fraction of equal bins, standard deviation of fraction of equal bins, mean fraction of unequal bins, standard deviation of fraction of unequal bins, mean fraction of equal bins with RAB, mean fraction of unequal bins with RAB, mean total fraction of genome affected by RAB per chromosome..

Supplemental Table 2: ANOVA results for all 3 technologies using the model: ks ~ individual+chromosome+individual:chromosome+replicate+Δbinlength, related to Figure 5. Columns from left to right are variables, degrees of freedom, sum squared, mean squared, F value, P value, P values adjusted by the Benjamini-Hochberg FDR method, technology, and reference.

Supplemental Table 3: Fractions of annotation space affected by RAB on a genome level, as an average across all samples, related to Figure 4. Left to right, columns are technology, annotation set, total number of annotations in set, fraction of annotations intersected with equal length bins, fraction of annotations intersected with unequal length bins, fraction of annotations intersected with equal length bins with RAB, fraction of annotations intersected with unequal length bins with RAB.

Supplemental Table 4: Genome Assembly Statistics, related to STAR Methods. Left to right, columns are assembly, number of contigs, N50, L50, number of single-copy genes, number of false duplications, fragment number, switch error rate, Hamming error rate, QV value, scaffold assembly gaps, and scaffold assembly gaps base pair size.

Supplemental Table 5: WebGestalt Over-representation analysis results, related to Figure 4. This table contains the raw results returned from WebGestalt after submitting RAB genes from each strata to each of several databases. Left to right, columns are stratum name, source database, an identified over-represented gene set, the description of the gene set, a link to the source of the gene set, the size of the set, the overlap between the submitted set and the identified set, the expected overlap, the enrichment ratio, the p-value, the false discovery rate (FDR), IDs of overlapping genes in the set, and the submitted gene IDs (ensembl gene IDs).

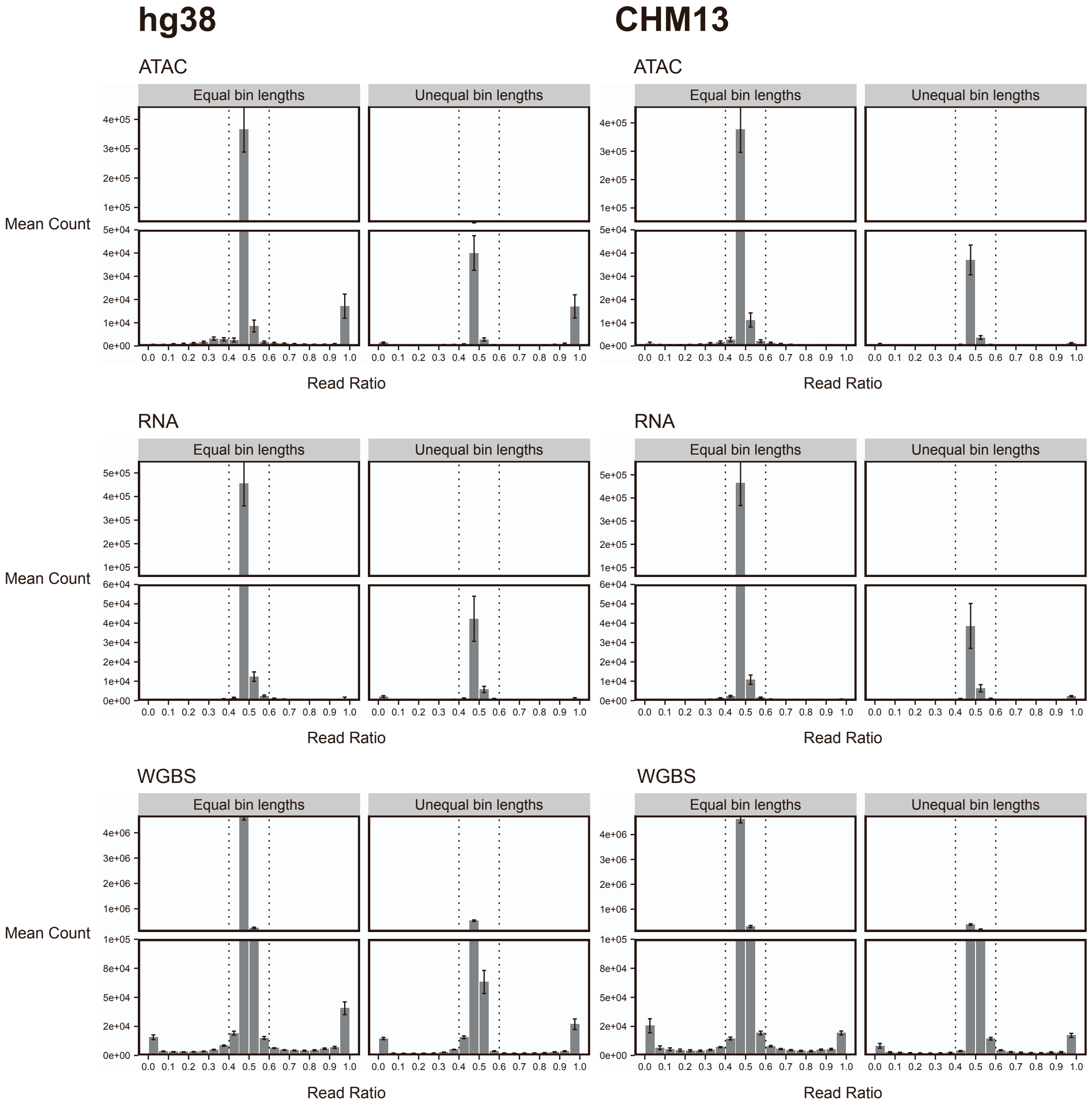

Supplemental Figure 1: <u>Distribution of reference fractions (Rf) across equal- and unequal-length bins for each sequencing technology and reference genome, related to Figure 4.</u> Each panel shows the count of bins (y-axis) as a function of reference fraction (Rf, x-axis), separated into equal- and unequal-length bins (left and right, respectively). Rows correspond to sequencing technologies (ATAC-seq, RNA-seq, and WGBS), and super-columns correspond to reference genomes (hg38 and CHM13). Dashed vertical lines indicate the Rf = 0.4 and 0.6 thresholds.

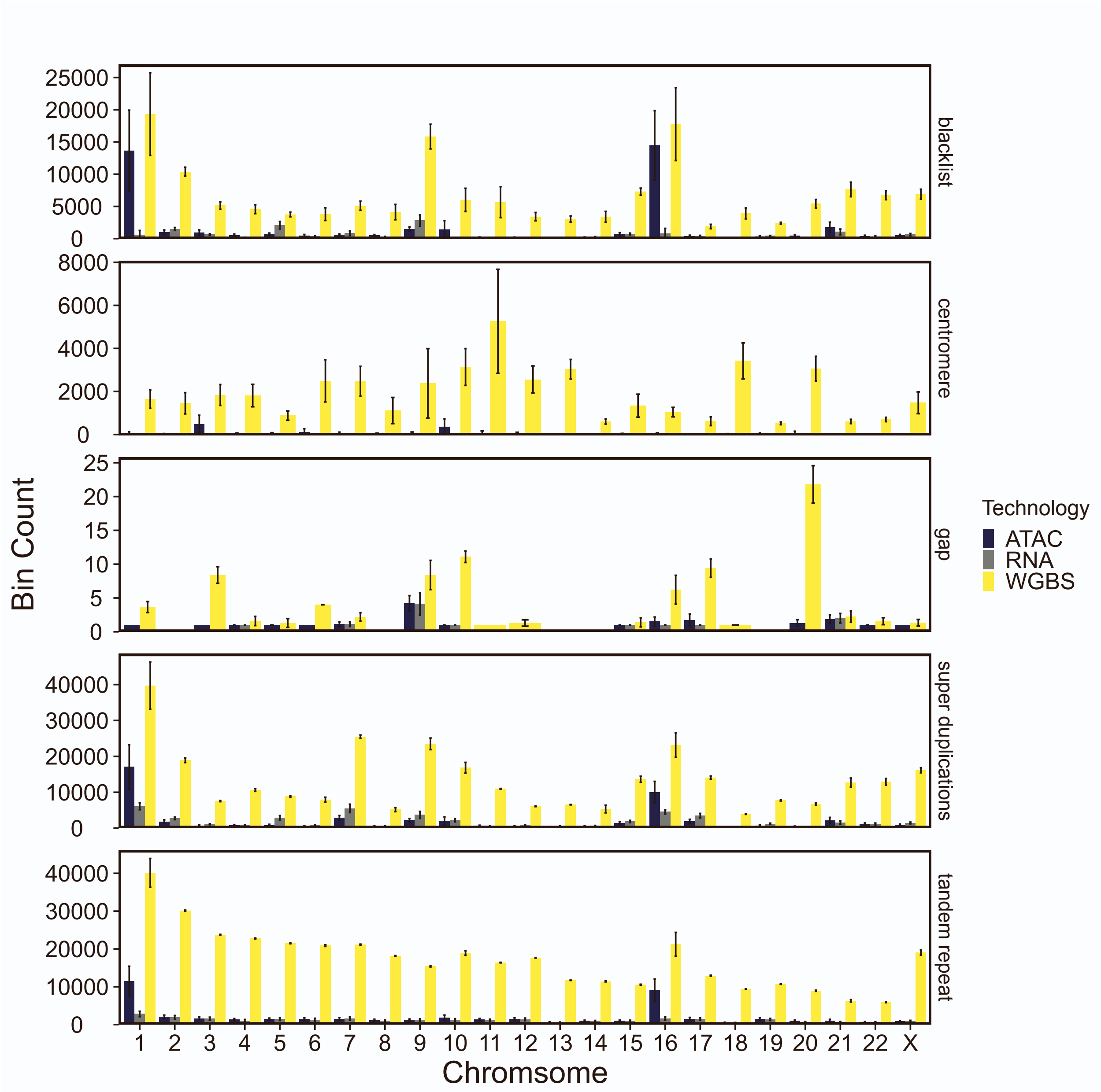

Supplemental Figure 2: Count of Bins Intersecting hg38 SV Annotations, related to Figure 4. This figure visualizes the mean number of bins (equal and unequal length) that intersect hg38 blacklist, centromere, gap, large duplication, and tandem repeat annotations. Data are separated by technology and plotted as the mean and standard deviation between individuals, parents, and replicates.

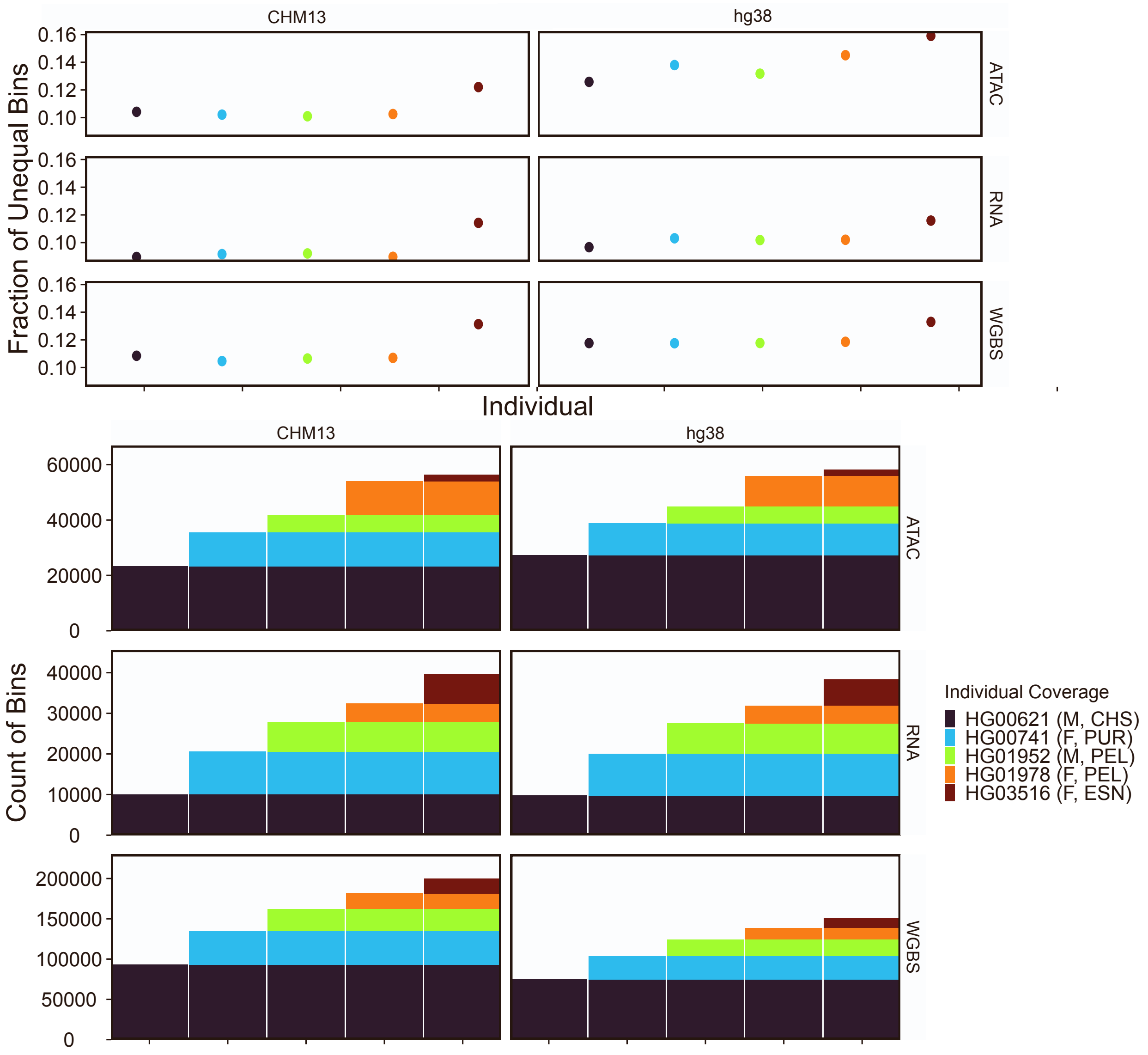

Supplemental Figure 3: Bias visualized across ancestry, related to Figure 5. A) The fraction of the total genome that has structural variation as measured by the fraction of unequal length bins across individuals relative to either CHM13 or hg38. B) Saturation analysis showing the cumulative number of new bins affected by reference alignment bias (RAB) as each additional individual is included, starting from HG00621. Individuals are ordered by ancestry: Han Chinese, Puerto Rican, Peruvian, and Esan.

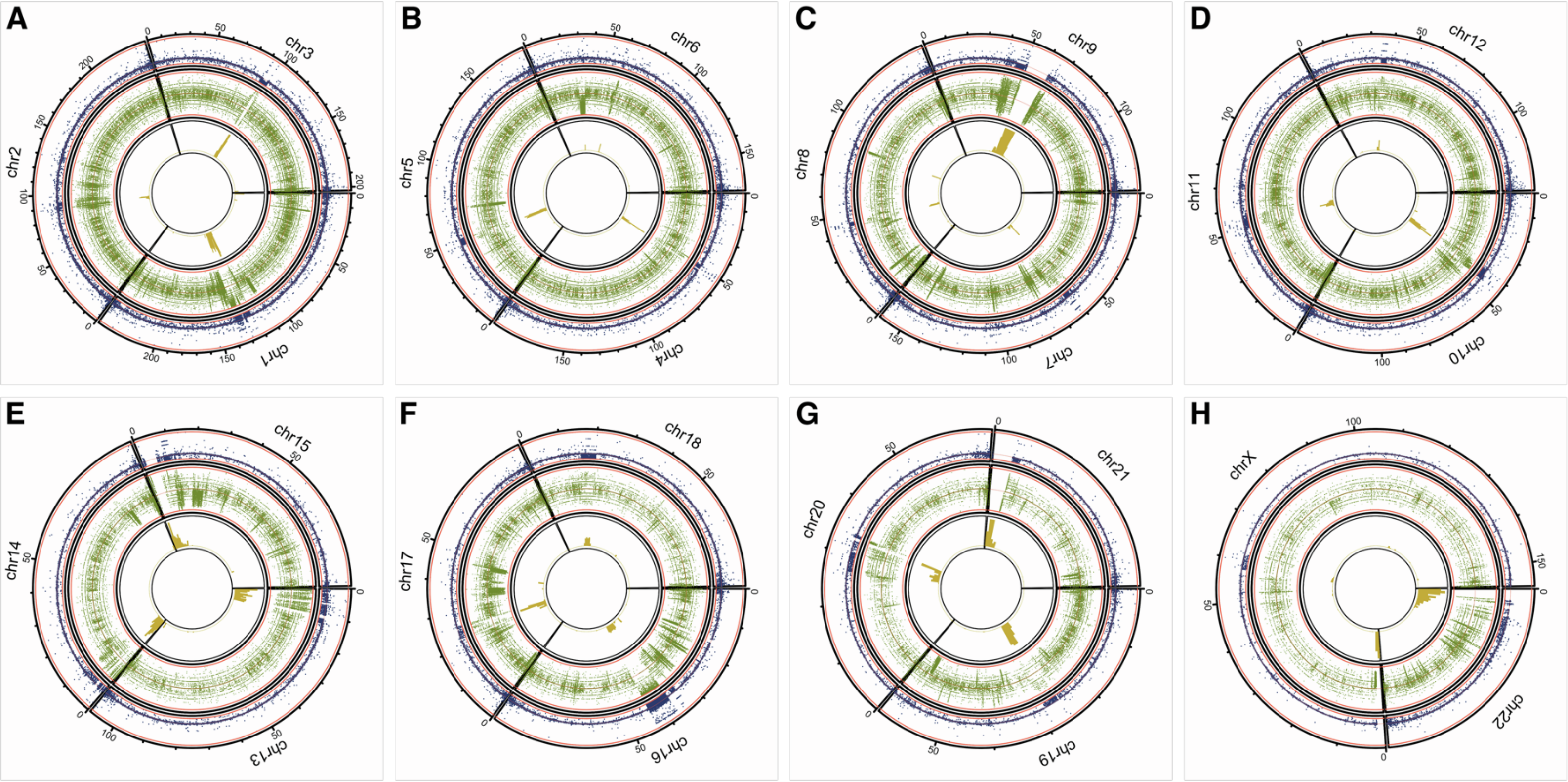

Supplemental Figure 4: Chromosome-level visualization of RNA-seq data aligned to HG01952 maternal assembly, related to Figure 6. Each chromosome is annotated by bins that did not liftover (inner track), bin reference fraction plotted for visualizations of variation from 0.5 (middle track), and bin length difference bounded from -250 to 1000 base pairs (outer track).

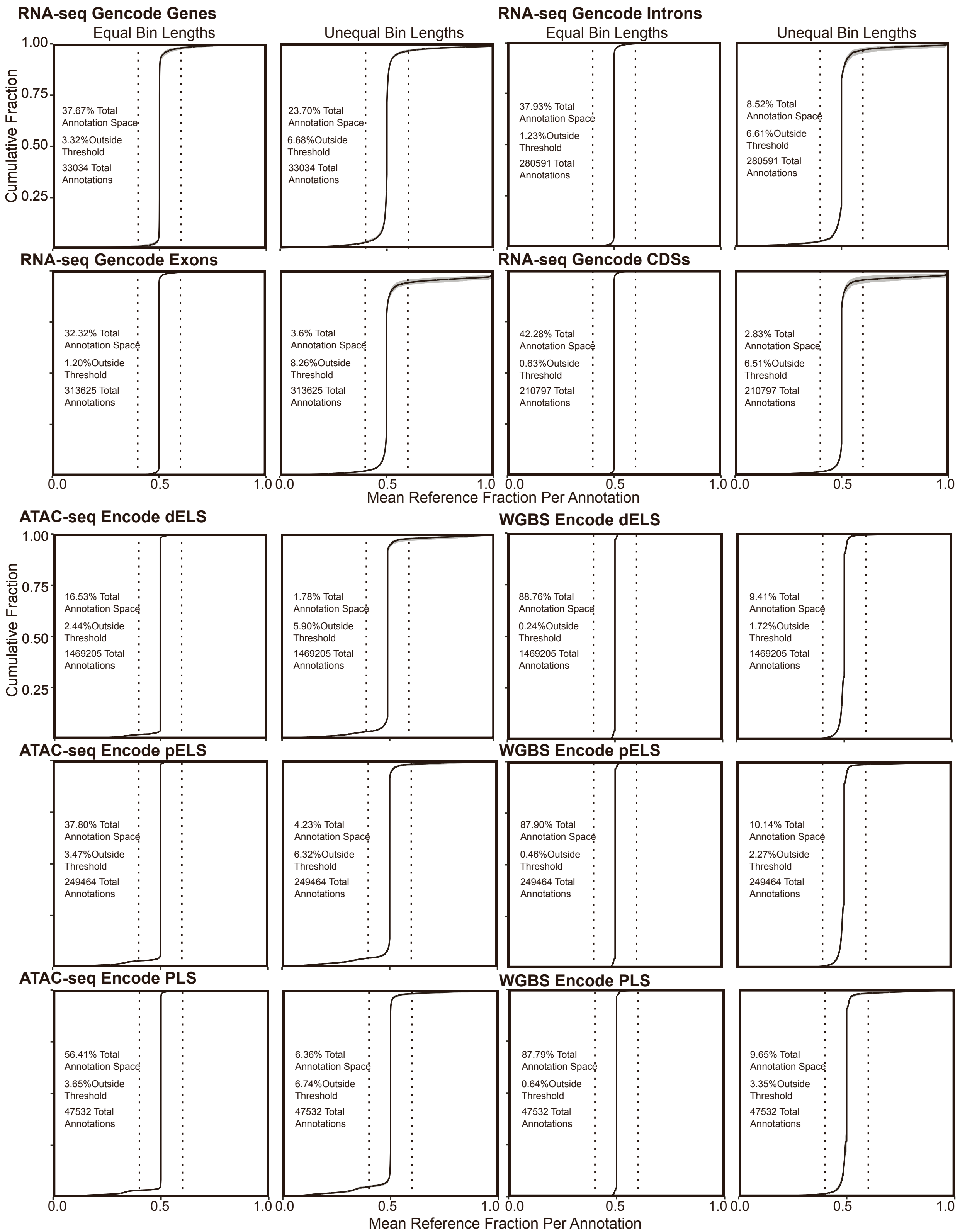

Supplemental Figure 5: Analysis of Reference Alignment Bias (RAB) Across Genome Annotations, related to Figure 4. This figure visualizes the cumulative distribution of mean reference fractions per annotation, assessing the extent of RAB within genomic data.

ATAC-seq and WGBS intersected bins were mapped to ENCODE promoter (PLS), distal enhancer (dELS), and proximal enhancer (pELS) annotations. RNA-seq intersected bins were mapped to GENCODE CDS, UTR, intron, and gene annotations. Data are split between annotations intersected with equal and unequal bin length Rf.

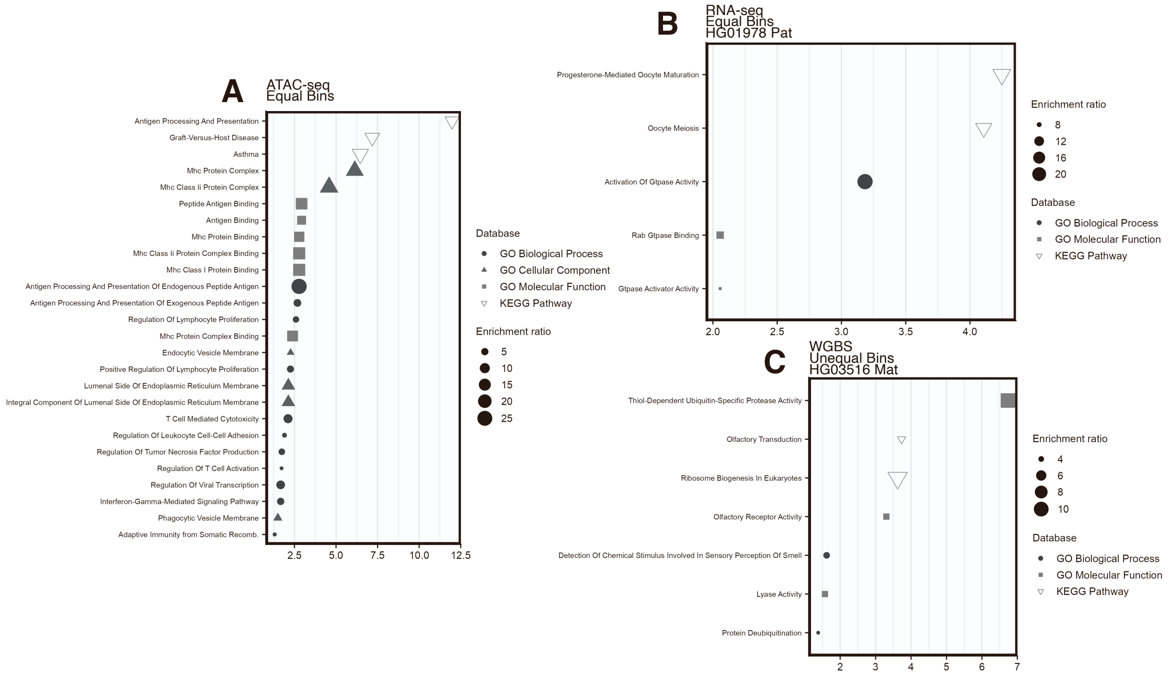

Supplemental Figure 6: Representative over-representation analysis (ORA) plots across various strata, related to Figure 4. x-axis: -log10(FDR). y-axis: top annotations per database with significance (FDR) < 0.05. Point size represents enrichment ratio, and point shape represents the source database of the annotation. **A)** ORA for all genes with ATAC-seq signal in equal-length bins. Most results are related to immune function, consistent with previous findings that show high genetic diversity in these genes. **B)** ORA for all genes with RNA-seq signal from the paternal haplotype of individual HG01978 falling into equal-length bins. **c:** ORA for all genes with whole genome bisulfite sequencing (WGBS) signal from the maternal haplotype of individual HG03516 falling into unequal length bins.

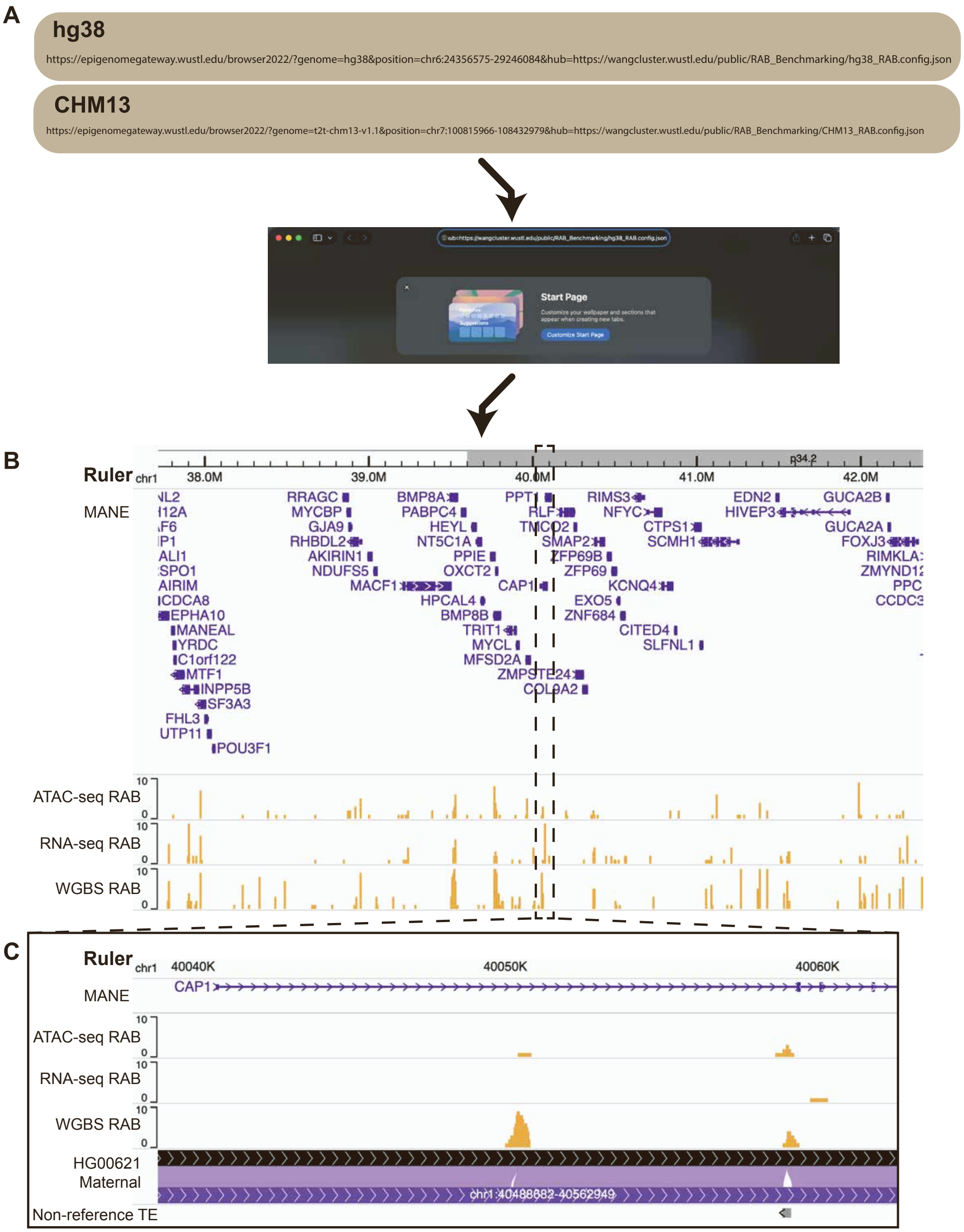

Supplemental Figure 7: Example of genome-wide RAB annotations and instructions for browser access, related to Figure 6. A) Instructions for accessing the genome-wide RAB annotations on the WashU Epigenome Browser. To view the tracks, click the provided link or paste the URL into a web browser. The hg38 annotations can be accessed at https://epigenomegateway.wustl.edu/browser2022/?genome=hg38&hub=https://wangcluster.wustl.edu/public/RAB_Benchmarking/hg38_RAB.config.json, and the CHM13 annotations at https://epigenomegateway.wustl.edu/browser2022/?genome=t2t-chm13-v1.1&hub=https://wangcluster.wustl.edu/public/RAB_Benchmarking/CHM13_RAB.confi

g.json. B) Example of a genomic region on chromosome 1 annotated for recurrent RAB across assays. C) Detailed view within this region showing the CAP1 gene, where two non-reference TE insertions are present, including a partial insertion found in a subset of samples and a complete Alu element, illustrating how sequence differences relative to the reference can contribute to local reference alignment bias.
